# Prediction of Long-Term Benefits of Inhaled Steroids by Phenotypic Markers in Moderate-to-Severe COPD: A Randomized Controlled Trial

**DOI:** 10.1371/journal.pone.0143793

**Published:** 2015-12-10

**Authors:** Jiska B. Snoeck-Stroband, Therese S. Lapperre, Peter J. Sterk, Pieter S. Hiemstra, Henk A. Thiadens, H. Marike Boezen, Nick H. T. ten Hacken, Huib A. M. Kerstjens, Dirkje S. Postma, Wim Timens, Jacob K. Sont, GLUCOLD Study Group

**Affiliations:** 1 Department of Public Health and Primary Care, Leiden University Medical Center, Leiden, The Netherlands; 2 Department of Pulmonology, Leiden University Medical Center, Leiden, The Netherlands; 3 University of Groningen, University Medical Center Groningen, Department of Epidemiology, Groningen, The Netherlands; 4 University of Groningen, University Medical Center Groningen, Department of Pulmonology, Groningen, The Netherlands; 5 University of Groningen, University Medical Center Groningen, Department of Pathology, Groningen, The Netherlands; 6 Department of Medical Decision Making, Leiden University Medical Center, Leiden, The Netherlands; New York University School of Medicine, UNITED STATES

## Abstract

**Background:**

The decline in lung function can be reduced by long-term inhaled corticosteroid (ICS) treatment in subsets of patients with chronic obstructive pulmonary disease (COPD). We aimed to identify which clinical, physiological and non-invasive inflammatory characteristics predict the benefits of ICS on lung function decline in COPD.

**Methods:**

Analysis was performed in 50 steroid-naive compliant patients with moderate to severe COPD (postbronchodilator forced expiratory volume in one second (FEV_1_), 30–80% of predicted, compatible with GOLD stages II-III), age 45–75 years, >10 packyears smoking and without asthma. Patients were treated with fluticasone propionate (500 μg bid) or placebo for 2.5 years. Postbronchodilator FEV_1_, dyspnea and health status were measured every 3 months; lung volumes, airway hyperresponsiveness (PC_20_), and induced sputum at 0, 6 and 30 months. A linear mixed effect model was used for analysis of this hypothesis generating study.

**Results:**

Significant predictors of attenuated FEV_1_-decline by fluticasone treatment compared to placebo were: fewer packyears smoking, preserved diffusion capacity, limited hyperinflation and lower inflammatory cell counts in induced sputum (p<0.04).

**Conclusions:**

Long-term benefits of ICS on lung function decline in patients with moderate-to-severe COPD are most pronounced in patients with fewer packyears, and less severe emphysema and inflammation. These data generate novel hypotheses on phenotype-driven therapy in COPD.

**Trial Registration:**

ClinicalTrials.gov NCT00158847

## Introduction

Accelerated decline in lung function is the hallmark of chronic obstructive pulmonary disease (COPD). In the past years, three studies provided evidence that the decline in postbronchodilator forced expiratory volume in one second (FEV_1_) can be reduced by long-term inhaled corticosteroid (ICS) treatment during one to three years of follow-up [1–3]. This was unexpected, since most large trials had not shown such benefit previously though, supported by, a recent study showed also deterioration of FEV_1_, after steroid withdrawal in COPD [4,5]. There appears to be considerable heterogeneity in treatment effects amongst COPD patients [1–3]. Hence, implementation of long-term ICS therapy in daily practice is difficult since this requires predictive patient characteristics to determine who should receive this long-term treatment and who should not [6,7].

Only a few clinical features have been reported that may predict ICS response in COPD. Reports on long-term predictors (years) are, however, sparse. Current smoking may reduce long-term ICS responses [8–11], as well as a higher number of packyears smoked, reflecting the cumulative effect of smoking on lung disease [8]. In daily practice, physicians are tempted to use lung function reversibility as a guide for prescribing ICS. A relatively large bronchodilator response was shown to predict short-term (months) ICS response [12–15]. However, conflicting results exist whether reversibility can predict long-term steroid effects [8,16,17]. Furthermore, many COPD patients demonstrate increased airway hyperresponsiveness, a factor related to accelerated FEV_1_-decline [18–20]. This possibly reflects a distinct phenotype of COPD patients that may exhibit an enhanced ICS treatment response [16,21,22].

The progressive decline of FEV_1_ in COPD is associated with an inflammatory process [2]. Persistent systemic inflammation is present in a subgroup of patients with COPD [23], and distinct inflammatory markers are likely to be incorporated in routine management of COPD patients [24]. Eosinophil and T-cell markers in the local airways may predict short-term lung function responses to ICS treatment in COPD [25,26]. However, to our knowledge, there are no data available on the predictive value of non-invasively obtained inflammatory cells for long-term effects of ICS treatment on the accelerated FEV_1_ decline in COPD.

We postulated that clinical, physiological and/or non-invasive inflammatory characteristics can serve as predictors of long-term beneficial effects of ICS on lung function decline in steroid-naive patients with moderately severe COPD. The present ***a priori*** planned analysis aims at hypothesis generation in order to reveal possible COPD phenotypes that will more likely benefit from anti-inflammatory therapy with respect to disease progression, a so far unexplored area in long-term studies with ICS treatment.

## Material and Methods

The present study focuses on detailed phenotyping of patients with moderate to severe COPD. A phenotype that is relevant for clinical practice, can be defined as ‘**a** single or combination of disease attributes that describe differences between individuals with COPD as they relate to clinically meaningful outcomes (symptoms, exacerbations, response to therapy, rate of disease progression, or death)’ [6,27]. For this purpose, 50 patients with COPD, who received 2.5 years of treatment with ICS or placebo in the GLUCOLD study (Groningen Leiden Universities and Corticosteroids in Obstructive Lung Disease) were included in the analysis. The original cohort also included patients who stopped ICS after 6 months and patients with the combination therapy of ICS and a long-acting β_2_-agonist. We excluded these patients in the current analyses because we focused on long-term effects of steroid therapy as such, without interference of additional effects by long-acting β_2_-agonists or withdrawal of stopping with inhaled corticosteroids.

The GLUCOLD study was a prospective longitudinal, randomized, double blind, placebo-controlled two-center trial. Patients were randomly assigned to receive either fluticasone propionate (500 μg bid) or placebo for 2.5 years. Measurements of symptoms and lung function were made every 3 months, whilst lung volumes, airway hyperresponsiveness, peripheral blood collection and induced sputum were obtained at baseline, 6 and 30 months. Detailed information on study medication, compliance and randomization procedure were published before [2,28] (S1 Protocol). Briefly, patients had irreversible lung function impairment that was compatible with the Global initiative for chronic Obstructive Lung Disease (GOLD) stages II and III [29], and had ≥ 10 packyears smoking. Asthma was excluded by a doctor’s diagnosis and by self-reported symptoms, treatment or diagnosis of asthma. Participants had not been treated with inhaled and oral corticosteroids within 6 and 3 months of trial entry respectively, and 47/50 patients were steroid-naive before entry of the study. Recruitment and follow-up was between 2000 and 2007. The vast majority was recruited from general practices and all patients had been clinically stable for at least 8 weeks before the measurements. The study was performed complying with the Helsinki declaration and approved by the medical ethical committees in Leiden (METC LUMC) and Groningen (METC UMCG) at the 6^th^ of April 2000 and all subjects gave written informed consent. This study was a fully investigator-initiated and -driven trial on ICS in COPD, registered at www.clinicaltrials.gov; identifier: NCT00158847. The trial was registered after enrolment of participants started, because at the time recruitment started registration was not yet obliged. The authors confirm that all ongoing and related trials for this drug/intervention are registered.

Spirometry was performed before and after inhalation of a short-acting bronchodilator, total lung capacity (TLC) and residual volume (RV) using a constant volume body plethysmograph, and airway hyperresponsiveness to methacholine with the two-minute tidal breathing method [28]. Patients were asked about dyspnea according to the American Thoracic Society guidelines [30] using the modified Medical Research Council (MRC) dyspnea scale (1 = no dyspnea to 5 = dyspnea at rest), and about the presence of chronic bronchitis (daily cough and sputum production for at least 3 months a year, for two consecutive years) [31]. Health status was measured using the St. George's Respiratory Questionnaire (SGRQ) [32] and the Clinical COPD Questionnaire (CCQ) [33].

Sputum induction and processing were performed according to a validated technique using the so called ‘full sample’ method [2,28]. Cell counts were expressed as both total and differential counts.

### Statistical analysis

For this study long-term (2.5-year) decline in postbronchodilator FEV_1_ was the main outcome parameter. Detailed information on power of the original GLUCOLD trial and compliance is published elsewhere [2]. Potential clinical predictor variables were ***a-priori*** selected according to a tailored strategy developed for small datasets [34]. Guidelines suggest that one candidate predictor can be studied for every 10 patients [35]. Since the current exploratory study population consisted of 50 patients, we selected 5 domains (with high baseline within-domain correlation) of potential baseline predictors: (1) packyears [8]; (2) presence of chronic bronchitis; (3) lung function (baseline FEV_1_, reversibility of FEV_1,_ [16], PC_20_ [18,22], RV/TLC); (4) TLCO; and (5) absolute and differential cell counts in induced sputum [26,36] (Table 1). Although continuous variables might provide more information we dichotomized variables based on median values in order to allow clinically meaningful interpretation of the results. A set of 2 linear mixed effect models [37] was used to identify predictors of long-term (2.5 years) ICS effect on change in postbronchodilator FEV_1_ over time [2,28]. For each potential predictor, the following variables were included in the model: time, treatment (placebo coded as 0 fluticasone as 1), predictor (reference coded as 0 index as 1), their interaction (treatment*predictor) and their interaction terms with time, using FEV_1_ as the dependent variable. Thus, the interaction term (treatment*predictor*time) reflects the additional value of the predictor to the effect of treatment with inhaled fluticasone compared to placebo on longitudinal changes in FEV_1_. An index category is defined relative to the median value, and represents the more favourable outcome by fluticasone. The reference category is complementary. Because of the limited number of patients, we tested these variables univariately in the model. The statistical analyses were performed with STATA 11.0 (StataCorp; College Station TX, USA). Differences at p-values ≤0.05 (tested 2-sided) were considered significant. The dataset is shown in S1 Table.

**Table 1:** A priori defined predictors. 5 Domains of potential predictors are listed in the table: (1) packyears; (2) presence of chronic bronchitis; (3) lung function (4) TLCO; and (5) absolute and differential cell counts in induced sputum.

Domains	Predictor
Smoking	Packyears
Chronic bronchitis	Presence of chronic bronchitis
Lung function	Baseline FEV_1_
	Reversibility of FEV_1_
	PC_20_
	TLC
	TLCO
Blood	IgE
	Differential cell counts
Sputum	Absolute count
	Differential cell counts

Definition of abbreviations: FEV_1_ = forced expiratory volume in one second; % pred = percentage of predicted value; PC_20_ methacholine = the provocative concentration of methacholine that causes a decrease in FEV_1_ of 20%; RV/TLC = residual volume/total lung capacity; TLC = total lung capacity; TLCO = diffusion capacity of the lung for carbon monoxide.

## Results

**Fig 1:**
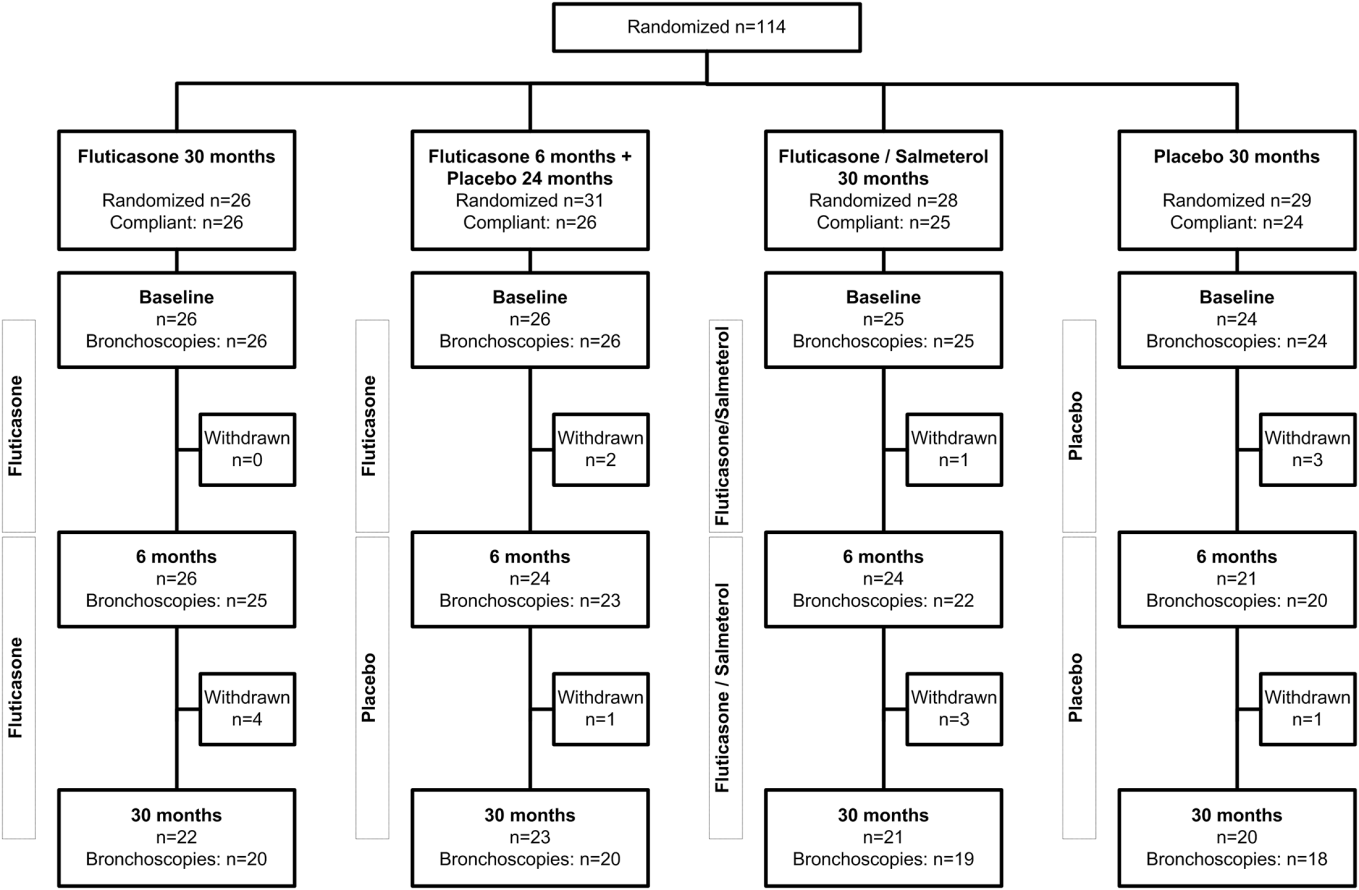
Study flow diagram. Total number of patients randomized and compliant (>70% medication use) per treatment group. At each stage of the study (0, 6 and 30 months) the numbers are listed of those who underwent bronchoscopy amongst the number of patients remaining in the study. We reproduced this flowchart from ‘Lapperre TS et al. (2009) Effect of fluticasone with and without salmeterol on pulmonary outcomes in chronic obstructive pulmonary disease: a randomized trial. Ann Intern Med 151: 517–527, with permission’.

Data from 26 patients with fluticasone and 24 patients with placebo treatment for 30 months were used for the present analysis (Table 2). In short, most patients were middle-aged males, two-thirds were current smokers, most heavily smoking for many years (Fig 1). The vast majority of patients could be classified as moderate-to-severe COPD, i.e. mean (SD) postbronchodilator FEV_1_ of 63±9% predicted. They were mildly impaired in health status, slightly reversible and had moderately severe bronchial hyperresponsiveness. Only 3 patients ever used inhaled corticosteroids before entry in the study.

**Table 2:** Patient characteristics at baseline. Characteristics of the study population per treatment group at baseline. Data represent mean (SD).

Characteristic	Fluticasone (n = 26)	Placebo (n = 24)
Age, yrs	62 (8)	59 (8)
Sex, m/f	23/3	20/4
Current smoking / ex-smoking	16/10	17/7
MRC dyspnea score ^‡^	2.6 (0.8)	2.7 (0.8)
SGRQ total score^†^	29 (13)	33 (19)
CCQ total score^§^	1.3 (0.6)	1.7 (1.3)
Postbr. FEV_1_, % pred	63 (8)	61 (8)
Reversibility, mL	3.2 (2.1)	3.6 (2.5)
Geom. mean PC_20_ methacholine (DD), mg/ml^║^	0.9 (2.8)	0.5 (2.4)
RV/TLC, %	47 (9)	47 (7)
TLCO, % pred	70 (20)	59 (16)
Median IgE (IQR), IU	28 (12–128)	51 (21–119)
Total number of sputum cells, x10^4^ cells/ml	175 (101–313)	168 (79–222)
Sputum cell percentages		
Neutrophils, %	66 (51–76)	72 (54–80)
Macrophages, %	29 (20–36)	22 (16–35)
Eosinophils, %	1.15 (0.3–2.2)	0.9 (0.3–2.2)
Lymphocytes, %	2.2 (1.3–3)	1.8 (1.3–3)

^║^PC_20_ methacholine is expressed as geometric mean (standard deviation in doubling dose (DD)).

^‡^range 1 to 5 (higher scores indicate more dyspnea);

^†^range 0 (best) to 100 (worst score);

^§^range 0 (best) to 6 (worst score).

Definition of abbreviations: postbr. = postbronchodilator; FEV_1_ = forced expiratory volume in one second; % pred = percentage of predicted value; PC_20_ methacholine = the provocative concentration of methacholine that causes a decrease in FEV_1_ of 20%; RV/TLC = residual volume/total lung capacity; TLCO = diffusion capacity of the lung for carbon monoxide.

### Predictors of changes in FEV_1_ decline by fluticasone treatment over 30 months

**Fig 2:**
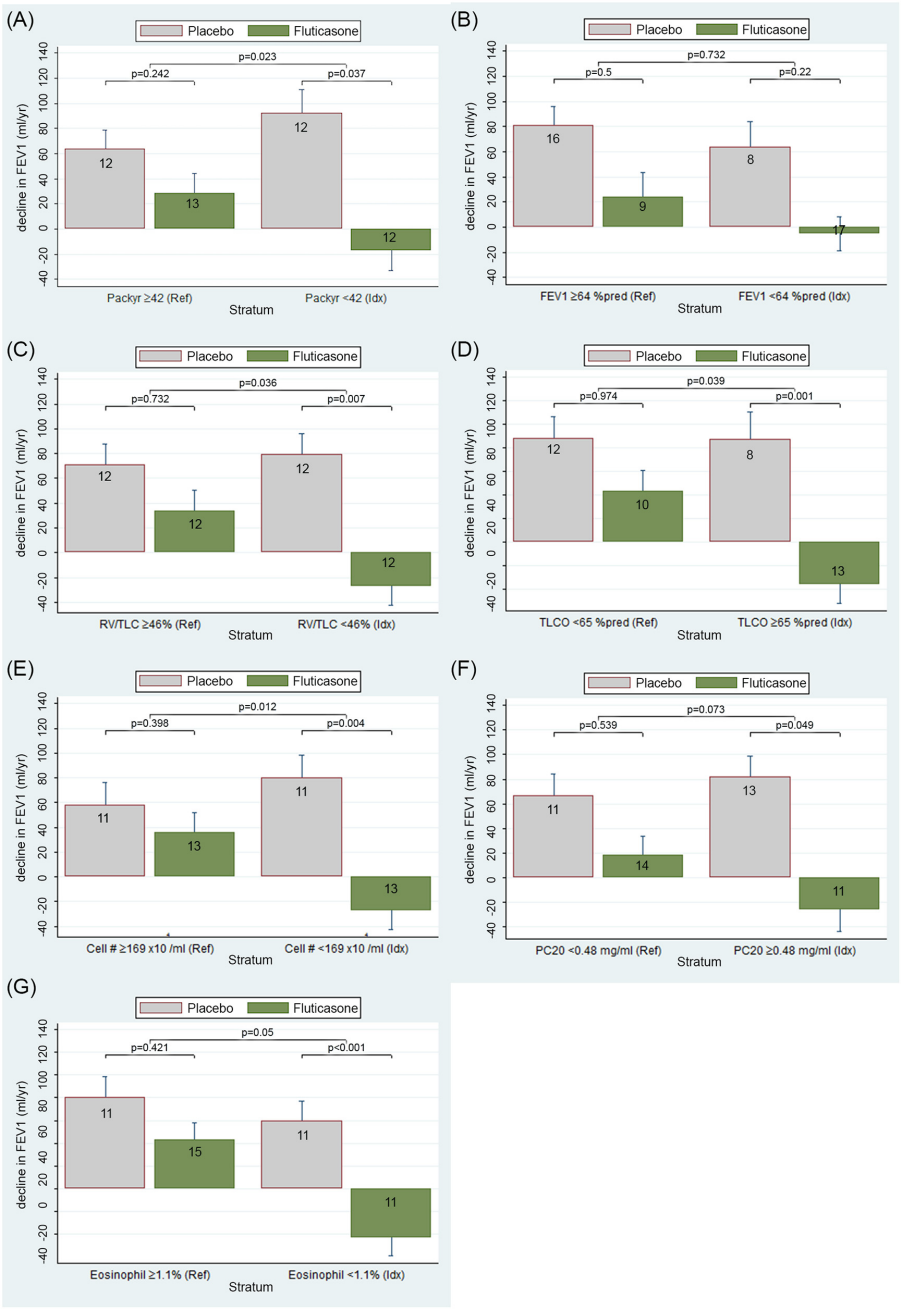
Long-term predictors of FEV_1_ decline by fluticasone treatment. (A) Prediction by packyears smoking of the decline of FEV_1_ by 2.5 year fluticasone treatment (ml) and placebo, stratified by median values of the predictor by 30 months treatment with fluticasone compared to placebo. ^#^Index category (Idx) is defined relative to median value, and represents the more favourable outcome by fluticasone. Reference category (Ref) is complementary. The group numbers of the patients are mentioned in each graph. For example: Fig 2A shows the decline in FEV_1_ in ml/yr on the Y axis for patients with many pack years and patients with few pack years with fluticasone or placebo treatment, respectively, on the X axis. All P values are based on the results of the linear mixed effects model. The treatment*time interaction term corresponding to the difference in decline in FEV_1_ between fluticasone and placebo in the low pack year stratum had a P value of 0.037. The interaction term (treatment*stratum*time) reflects the additional effect of pack years smoking stratum to the effect of treatment with inhaled fluticasone compared to placebo on longitudinal changes in FEV_1_. The corresponding P value for pack years smoking is 0.023. A favourable effect on decline in FEV_1_ would be a decrease in decline caused by inhaled corticosteroids. The figure shows that a lower number of packyears (= index category) decreases the decline in FEV_1_ significantly. (B) Prediction by baseline FEV_1_ of the decline of FEV_1_ by 2.5 year fluticasone treatment (ml) and placebo, stratified by median values of the predictor by 30 months treatment with fluticasone compared to placebo. (C) Prediction by RV/TLC of the decline of FEV_1_ by 2.5 year fluticasone treatment (ml) and placebo, stratified by median values of the predictor by 30 months treatment with fluticasone compared to placebo. (D) Prediction by TLCO of the decline of FEV_1_ by 2.5 year fluticasone treatment (ml) and placebo, stratified by median values of the predictor by 30 months treatment with fluticasone compared to placebo. (E) Prediction by total number of cell counts in induced sputum of the decline of FEV_1_ by 2.5 year fluticasone treatment (ml) and placebo, stratified by median values of the predictor by 30 months treatment with fluticasone compared to placebo. (F) Prediction by PC_20_ methacholine of the decline of FEV_1_ by 2.5 year fluticasone treatment (ml) and placebo, stratified by median values of the predictor by 30 months treatment with fluticasone compared to placebo. (G) Prediction by percentage of eosinophils in sputum of the decline of FEV_1_ by 2.5 year fluticasone treatment (ml) and placebo, stratified by median values of the predictor by 30 months treatment with fluticasone compared to placebo.

Long-term predictors of ICS effects on FEV_1_ decline are shown in Table 3 and Fig 2. Fluticasone improved the accelerated decline in FEV_1_ significantly more in patients with fewer packyears than those with more packyears smoked, the difference being +75 ml/yr (95%CI +10/+139, p = 0.023; Fig 2A). Baseline FEV_1_ was not a predictor of the ICS effect on FEV_1_ decline (Fig 2B). Furthermore, fluticasone improved FEV_1_ decline significantly more in patients with lower RV/TLC than those with higher RV/TLC (+69 ml/yr, 95%CI +4/+134, p = 0.036) (Fig 2C). Finally, higher CO-diffusion capacity (+78 ml/yr, 95%CI +4/+153, p = 0.039) (Fig 2D) and lower total sputum cell counts (+85 ml/yr, 95%CI +19/+152, p = 0.012) (Fig 2E) were also predictors of the ICS effect on FEV_1_ decline. Comparable but non-significant trends were found in patients with a shorter duration of smoking (Table 4), less airway hyperresponsiveness (Fig 2F) and a lower percentage of eosinophils in induced sputum (Fig 2G). All other variables tested, i.e. reversibility, IgE, circulating blood eosinophils, sputum neutrophils, lymphocytes and macrophages were not independent predictors of ICS-induced alternation of FEV_1_ decline.

**Table 3:** Mean values of predictors per stratum per treatment.

	Fluticasone (n = 26)		Placebo (n = 24)	
Predictor	Index^#^	Reference^⊥^	Index	Reference
Median smoking history (IQR), packyears	31 (25–34)	51 (47–62)	35 (31–41)	55 [47–63]
Median number of years smoking, (IQR), yrs	37 (32–39)	47 (45–54)	38 (33–40)	48 (46–54)
Postbr. FEV_1_, % pred	69 (3.4)	54 (6.3)	69 (3.4)	58 (6.1)
Reversibility in FEV1, % predicted	10.3 (2.3)	4.1 (2.4)	11.0 (2.9)	3.9 (2.6)
Geom. PC_20_ methacholine (DD), mg/ml^║^	2.3 (1.6)	0.05 (1.5)	2.4 (2.3)	0.10 (1.6)
RV/TLC, %	39 (5.1)	55 (8.2)	41 (4.1)	51 (3.3)
TLCO, % pred	83 (17.1)	54 (7.9)	74 (7.7)	48 (12.5)
Blood eosinophils, %	3.7 (1.3)	3.8 (1.5)	1.2 (2.0)	1.2 (1.7)
Gmean sputum total cell count (GSD), 10^4^/ml^‡^	62 (2.1)	272 (1.6)	86 (1.7)	392 (1.8)
Sputum cell percentages				
Neutrophils, %	51 (1.3)	80 (1.1)	51 (1.3)	79 (1.1)
Macrophages, %	16 (1.6)	40 (1.4)	17 (1.3)	39 (1.3)
Eosinophils, %	0.3 (2.4)	2.4 (1.6)	0.4 (2.4)	2.4 (1.7)
Lymphocytes, %	2 (1.2)	4 (1.2)	2 (1.3)	4 (1.3)

^#^Index category is defined relative to median value, and represents the more favourable outcome by fluticasone. For example: decline of FEV_1_ by fluticasone is diminished (= favourable outcome) in patients with a lower number of packyears (= index category).

^⊥^Reference category is complementary to the index category.

Values are mean (standard deviation (SD)), unless stated otherwise.

^║^PC_20_ methacholine is expressed as geometric mean (standard deviation in doubling dose (DD)),

^‡^sputum cells as Geometric mean (GSD).

Definition of abbreviations: postbr. = postbronchodilator; FEV_1_ = forced expiratory volume in one second; % pred = percentage of predicted value; PC_20_ methacholine = the provocative concentration of methacholine that causes a decrease in FEV_1_ of 20%; RV/TLC = residual volume/total lung capacity; TLCO = diffusion capacity of the lung for carbon monoxide.

**Table 4:** Predictors of attenuation of long-term FEV_1_ decline by fluticasone treatment.

Predictor	Index category^#^	Difference in change of FEV_1_ml/yr	95% CI	P value
Packyears, yrs	<42	+75	+10/+139	0.023
Number of years smoking, yrs	<42	+63	-0.7/+127	0.052
Postbr. FEV_1_, % pred	<64	+12	-56/+81	0.73
Reversibility in FEV1, % pred.	>7.4	-16	-80/+47	0.62
PC_20_ methacholine, mg/ml	≥0.48	+60	-6/+126	0.074
RV/TLC, %	<46	+69	+4/+134	0.036
TLCO, % pred	≥65	+78	+4/+153	0.039
Blood eosinophils, %	<2.4	-17	-0.83/+49	0.61
Sputum total cell count, 10^4^/ml	<169	+85	+19/+152	0.012
Sputum cell percentages				
Neutrophils, %	<69	+44	-23/+111	0.20
Macrophages, %	>25	-34	-102/+34	0.33
Eosinophils, %	<1.1	+66	-0.2/+132	0.051
Lymphocytes, %	<3	-7	-74/+61	0.85

Definition of abbreviations: postbr. = postbronchodilator; FEV_1_ = forced expiratory volume in one second; % pred = percentage of predicted value; PC_20_ methacholine = the provocative concentration of methacholine that causes a decrease in FEV_1_ of 20%; RV/TLC = residual volume/total lung capacity; TLCO = diffusion capacity of the lung for carbon monoxide.

^#^Index category is defined relative to median value, and represents the more favourable outcome by fluticasone. Reference category is complementary. The interaction term (treatment*stratum*time) reflects the additional effect of predictor variables to the effect of treatment with inhaled fluticasone compared to placebo on longitudinal changes in FEV_1_. The corresponding P values for predictor variables are reported in Table 4. A favourable effect on decline in FEV_1_ would be a decrease in decline caused by inhaled corticosteroids.

## Discussion

This study demonstrates that clinical and inflammatory phenotypes can partly predict efficacy of long-term inhaled corticosteroid use with respect to preventing FEV_1_-decline in steroid-naive patients with moderate to severe COPD. Patients with fewer packyears smoked, preserved diffusion capacity of the lung, limited hyperinflation, and lower number of total cells in induced sputum benefitted most from inhaled corticosteroids. These data indicate that features of less advanced disease, i.e. fewer signs of emphysema and less hyperinflation, as a marker of small airway dysfunction, predict a better course of lung function with treatment of ICS in patients with moderate to severe COPD. This suggests that COPD patients expressing milder and/or earlier stages of the disease, or a subtype of COPD patients in whom airway obstruction is accompanied by less severe emphysema or inflammation can benefit from anti-inflammatory therapy, which favors a differential approach in the treatment of COPD.

The long-term efficacy of fluticasone in patients with fewer packyears is in line with a previous study on long-term effects of budesonide in patients with mild COPD who continued smoking [8]: patients with <36 packyears had more benefit from budesonide. The present study extends these findings by showing, for the first time, that less impaired diffusion capacity and less hyperinflation predict a better effect of fluticasone therapy on FEV_1_-decline. It has been shown previously that short-term treatment with a combination of inhaled corticosteroids and long-acting beta_2_-agonist reduces lung hyperinflation in patients with severe COPD [38]. We here show that less hyperinflation itself may signify a beneficial ICS induced FEV_1_ response as well. Together our data suggest that ICS treatment in patients with airway involvement rather than parenchymal involvement provides beneficial effects, a finding supported by recent concepts [39].

Remarkably, in our study long-term effects on decline in FEV_1_ were more pronounced in patients with lower number of sputum cell counts. Notably, sputum eosinophil counts and circulating blood eosinophils were not significantly predictive of steroid-effects. This seems to contrast with previous studies showing positive effects of ICS on FEV_1_ in patients with more eosinophilic inflammation [25,26,36]. Eosinophilic inflammation may be an important biomarker pointing to a specific clinically relevant phenotype. Patients with a higher blood eosinophil count may have increased steroid responsiveness [26,40,41]. However, in our study, sputum and blood eosinophils were both not predictive of the effect of fluticasone on the decline in FEV_1_. This might be due to differences in patient selection. Most of the patients in our study were steroid naïve and had less severe COPD as based on a higher level of lung function and low exacerbation rates. Other reasons may be that the predictive effects of eosinophils are more pronounced with exacerbations than with decline in FEV_1_ [42]. Also, there may be different effects of inhaled corticosteroids versus oral steroids, even though a treatment regimen with inhaled steroids based on sputum eosinophils also let to an improved disease outcome in COPD [25]. In addition, the percentage of eosinophils in our patients is somewhat lower compared to results from the ECLIPSE Study where 37% of COPD patients had blood eosinophil counts above 2. In this study only 5 patients had more than 3% sputum eosinophils. The fact that most patients at baseline had low eosinophilic inflammation may (partly) explain the lack of predictive value of sputum eosinophils. In addition, a recent study showed that blood eosinophils were not associated with FEV_1_ decline [42]. Our methods for sputum analyses are well validated, and we expect this had no significant impact on the number of sputum eosinophils. Taken together, our results consistently point towards the notion that the beneficial effects of long-term treatment with ICS can be predicted by clinical and inflammatory features in particular phenotypes suggestive of less advanced COPD and/or a type of COPD with less emphysema. Future studies are needed to establish whether e.g. composite scores of these characteristics provide an even better prediction, but this requires a larger study group in view of the size of subgroups.

Obviously, there are some limitations to our study. The limited sample-size merely allowed univariate analyses of the predictors. Notwithstanding this, the fact that the long-term treatment effects on FEV_1_ decline over 2.5 years were consistently predicted by a number of indicators of less advanced disease supports the plausibility of our findings. Whereas, this assumes that the tested variables are independent, very often this strict assumption is not met with real world data, where even variables from different disease dimensions are (at least weakly) correlated [43]. In addition, application of a correction for multiple testing comes with the price of potentially neglecting real associations (type two error). Since this is a hypothesis generating study, we reported the exact P-values, thereby allowing the reader to apply a correction for multiple testing when he/she finds this appropriate [43]. We did not assess different cut-off values that are known in clinical research in order to avoid multiple testing and data driven decisions [34]. Instead, we used specific cut-off values based on medians of predictor variables. ***A priori*** we had decided to use the median for a couple of reasons. First, for many predictors there is no consensus on cut-off values or a cut-point is not established at all. Analyzing all different cut-points would increase the number of analyses. Secondly, by choosing the median values we argued that it would be a reasonable approach to dichotomize variables into “relatively low and high values” with similar group sizes as a first step. In general, future hypothesis testing studies may then reveal specific cut-off values of those variables that were predictive of the outcome in the current study. Dichotomizing a variable introduces loss of information [44]. However, by including continuous variables in the model one assumes a linear relationship, which is often not the case. Furthermore, if a strong association between continuous predictor and outcome exists, it is likely that it will also be captured with the dichotomized variable, despite lower power. In our view significant results based on analyses with dichotomized variables made the study more robust. Although we showed a significantly larger treatment effect on FEV_1_ decline in the index as compared to the comparator category for a number of indicators, we cannot exclude some efficacy of inhaled corticosteroids in the latter given the limited statistical power of our study.

How can we interpret these data? Glucocorticosteroids are effective in COPD in the COPD phenotype of frequent exacerbators by reducing exacerbations and current guidelines recommend ICS for this COPD phenotype. Hence, the question is not whether ICS can be effective in COPD, but which phenotypes are sensitive and why? The big trials during the past decade have not observed efficacy of ICS on the decline of FEV_1_ in COPD [4]. However, these results were based on results of patients with less detailed characterization. In contrast, the present study used extensive characterization, which allowed detailed phenotyping. Our results suggest that certain phenotypes of COPD, including patients with fewer packyears smoked, less hyperinflation, better CO-diffusion capacity and lower inflammatory load are characterized by preserved, long-term sensitivity to ICS. This is in keeping with the observations that patients with more advanced, severe COPD are less responsive to both the [45] introduction and [5] withdrawal of inhaled steroids.

There are multiple distinct mechanisms that contribute to steroid sensitivity and resistance. Altered regulation of the expression and activation of the transcription factor activator protein-1 (AP-1) in asthma is associated with steroid resistance [46]. In COPD, advanced oxidative stress reduces histone deacetylase-2 (HDAC2) so that inflammation might become less sensitive to the anti-inflammatory effects of steroids [47]. Our observation also suggests that fewer packyears rather than current smoking status predict a better efficacy of long-term inhaled steroids on decline of FEV_1_. This suggests that a relatively higher cumulative amount of smoking contributes to the irreversibility of the damage, and unresponsiveness to ICS. In addition, our findings are in keeping with the notion that advanced inflammation in COPD with mechanical changes of the airways, as indirectly reflected by hyperinflation, may contribute to relative glucocorticosteroid resistance. Furthermore, hyperinflation may influence progression of the disease, being associated with frequency of exacerbations, mortality and a rapid decline in FEV_1_ [48].

Whereas steroid resistance may be more pronounced in advanced disease, FEV_1_ decline is most progressive in early disease, pointing to the relevance of intervention in an early phase [49,50]. In addition, development of different phenotypes of chronic lung diseases start already in childhood or even in utero [51]. Taken together, our data raise the hypothesis that treatment with ICS at an earlier stage of the disease or in patients with less emphysema and/or less hyperinflation, may provide a window of opportunity to slow down the progression of the disease. This earlier stage is likely not reflected by FEV_1_ itself, since the European Respiratory Society Study on Chronic Obstructive Pulmonary Disease (EUROSCOP) study in mild COPD did not show a beneficial effect of 2-year ICS treatment on FEV_1_ decline [52].

These data have implications for clinical care. We studied COPD patients with mainly moderate disease severity. Even though most studies on treatment predictors have been performed in more severe disease, the majority of patients in day-to-day care have less advanced COPD. It can be questioned whether assessment of symptoms and FEV_1_ solely suffices in early stages [53]. Subgroups of patients with COPD such as those with more emphysema have been shown to display accelerated decline of FEV_1_ [54,55]. Even before emphysema is manifest, destruction has already started by loss of terminal bronchioles [56]. Our study generates the hypothesis that treating these patients as early as possible before damage has occurred could be of major importance for slowing down the disease progression.

Taken together, our data raise the point that certain subgroups such as patients with less advanced disease or those with a particular sub-phenotype of COPD with less emphysema and less small airway dysfunction might benefit from a differential therapeutic approach. This novel hypothesis requires external validation in larger subsets of patients with moderate to severe COPD, before our results can be generalized into daily practice. This approach would allow extending current recommendations in COPD guidelines by offering a promising perspective for subgroups of patients with COPD towards the potential of slowing down the progression of the disease.

## Supporting Information

S1 CONSORT Checklist (PDF)

S1 Protocol (PDF)

S1 Protocol Amendment (PDF)

S1 Table Dataset. http://figshare.com/s/c279dbb0639711e5aece06ec4b8d1f61
(XLS)
