# Supplementary material for: Prediction of Long-Term Benefits of Inhaled Steroids by Phenotypic Markers in Moderate-to-Severe COPD: A Randomized Controlled Trial
Source: PLoS One. 2015 Dec 10;10(12):e0143793. doi: 10.1371/journal.pone.0143793 (PMC4699453; doi:10.1371/journal.pone.0143793)
Supplement: S1 Protocol — (PDF) [file pone.0143793.s002.pdf]

Research Protocol

Modification of disease outcome in COPD

Shortterm versus longterm treatment with inhaled corticosteroids, either or not combined with a long-acting ß$_2$-agonist

The GLUCOLD Study Group

Groningen Leiden Universities Corticosteroids in Obstructive Lung Disease

Dept. of Pulmonology
University Hospital Groningen
P.O. Box 30.001
NL-9700 RB Groningen
The Netherlands
tel: +31 50 361 3532
fax: +31 50 361 9320
email: d.s.postma@int.azg.nl

Dept. of Pulmonology
Leiden University Medical Centre
P.O. Box 9600
NL-2300 RC Leiden
The Netherlands
tel: +31 71 526 3578
fax: +31 71 515 4691
email: psterk@pulmonology.azl.nl

Version: GLUCOLD6.prt (March, 2000)

Grant:
- Strategisch Onderzoek (SO), Gebiedsbestuur Medische Wetenschappen, programma Chronisch Zieken, NWO 1998 (940-35-033)
- Astma Fonds (93.96.3)
- Medische Faculteiten: LUMC and AZG/RUG
- Glaxo Wellcome, Nederland

The GLUCOLD Study Group

Groningen Leiden Universities Corticosteroids in Obstructive Lung Disease

Groningen

Dept. Pulmonology:	D.S. Postma, H.A.M. Kerstjens, N.H.T. ten Hacken, Th.W. van der Mark, M.S. van Maaren
Dept. Pathology:	W. Timens, B.W.M. Willemse
Dept. Epidemiol & Stats:	H.M. Boezen, J.P. Schouten
Dept. General Practice:	T. van der Molen
Dept. Allergology:	H.F. Kauffman

Leiden

Dept. Pulmonology:	P.J. Sterk, L.N.A. Willems, J. Stolk, P.S. Hiemstra, W.I. de Boer, K.F. Rabe, T.S. Lapperre
Dept. Pathology (AZN):	J.H.J.M. van Krieken
Dept. General Practice:	G.H. de Bock, H.A. Thiadens, J.B. Stroband
Dept. Med. Decision Making:	J.K. Sont

Introduction

Chronic obstructive pulmonary disease (COPD) is a major cause of morbidity and the fifth leading cause of mortality worldwide [Siafakas 1995, Postma 1998]. The WHO organisation has foreseen that COPD mortality will be at the third ranking, after cancer and cardiovascular mortality, by the year 2020. This calls for adequate intervention in the progression of disease and, above all, a better understanding of the pathogenesis and pathophysiology of COPD.

Current knowledge shows that the disease is characterized by progressive and largely irreversible airways obstruction [Postma 1998]. This is demonstrated by an accelerated decline with age of FEV_1 (forced expiratory volume in 1 second), associated with symptoms of dyspnoea at rest or on exertion. The disease leads to impaired quality of life, disablement and eventually death. The pathogenesis of COPD is closely associated with cigarette smoking. Risk factors for the development of COPD in smokers are continuation of smoking, low FEV_1, and increased airway hyperresponsiveness [Tashkin 1996]. Furthermore, it has recently been reported that elevated serum IgE and peripheral blood eosinophilia are associated with a more rapid decline of FEV_1 in smokers [Villar 1995]. It appears that the susceptibility, development, clinical course and prognosis of COPD are at least partly determined by host characteristics.

The pathology of COPD is heterogeneous [Saetta 1994, Postma 1998]. It can include (acute and chronic) inflammation within the small airways as well as features of emphysema. The bronchiolar and peribronchiolar inflammation in smokers with COPD is characterized by *e.g.* increased numbers of epithelial mast cells and macrophages [Grashoff 1997], elevated numbers of neutrophils and of $CD3^+$ T lymphocytes with an increase in the $CD8^+$ subset [Saetta 1998], and increased expression of growth factors, such as TGFß (transforming growth factor beta) [de Boer 1998]. It is important to notice that some of these pathological features can be confirmed in bronchial biopsy specimens as obtained from the large airways [O'Shaughnessy 1997]. The current working hypothesis is that this inflammatory response not only leads to swelling and structural changes of the airway walls (fibrosis, smooth muscle growth) [Saetta 1998], but also to destruction of peribronchiolar alveolar attachments and parenchymal destruction by an imbalance of local proteases (such as neutrophil elastase) and their inhibitors (such as SLPI) [Thurlbeck 1990]. As this imbalance and the resulting destruction are not completely explaining the development of emphysema, it can be postulated that qualitative or quantitative defects in local tissue repair are also important in COPD

(emphysema) patients. Tissue repair as a form of wound healing is regarded as an inflammatory process, in which inflammatory cells play a major regulating role. Consequently, long-term anti-inflammatory therapy can be expected to reduce not only damaging inflammation, but also the local tissue repair process [Timens 1997].

In addition to affecting tissue repair, steroids may also affect the local production of glutathione (GSH), a major anti-oxidant in the lung. Whereas cigarette smoke increases GSH both *in vivo* and *in vitro*, the steroid dexamethasone was found to decrease GSH production in cultured airway epithelial cells (Rahman 1998). Finally, steroids may also increase the anti-inflammatory screen in the lung by increasing the production of the serine proteinase inhibitor secretory leukocyte proteinase inhibitor (SLPI), as has been shown *in vitro* (Abbinantie-Nissen, 1995). These observations have not yet been confirmed by *in vivo* studies, since treatment of patients with chronic bronchitis and emphysema by inhaled steroids did result in significant increases in sputum SLPI levels, just failing to reach statistical significance (Llewellyn-Jones 1996).

Based on the above, it may not be surprising that the benefits of currently available therapeutic interventions in COPD are limited [Siafakas 1995, Postma 1998]. There is no doubt that smoking cessation remains the best option, so far being the only intervention that leads to a reduction in the annual decline in FEV_1 [Tashkin 1996]. Interestingly, such improvement is partly predicted by the level of airway hyperresponsiveness to methacholine [Tashkin 1996], which is thought to be determined by airway remodelling next to inflammatory cell activity [Reiss 1996].

The other intervention which has been postulated to reduce disease progression is anti-inflammatory treatment by oral or inhaled corticosteroids [Postma 1988,1991,1998]. Indeed, there is evidence from prospective studies that regular therapy with inhaled corticosteroids leads to an improvement in FEV_1 within a 6-months period [Dompeling 1992]. However, such treatment does not seem to improve the annual decline in FEV_1 during long-term follow-up [Renkema 1996], although there seems to be clinical benefit in terms of symptoms, exacerbation rate and exercise capacity [Paggiaro 1997]. The transient benefits on FEV_1 have recently been confirmed in two large-scale multicentre European trials (still to be published: EUROSCOP, ISOLDE), in which inhaled steroid treatment in patients with COPD led to an initial 3-6 months of improvement in FEV_1, which was maintained during the following 2.5 years of treatment. However, the subsequent course of FEV_1 (annual decline) after the first 3-6

months remained unaltered. This is indicative of transient benefits of inhaled steroids in COPD, predominantly during the first months of treatment. Using oral steroid therapy in COPD it has been shown that the greatest response occurs in those patients with eosinophilic inflammation within the airways. This suggests that steroids might reduce some of the features of acute airways inflammation in COPD, whereas they may not influence the structural changes within the airways. As indicated above, it may even be postulated that steroids can have deleterious effects on chronic inflammation [Timens 1997], *e.g.* by limiting neutrophil apoptosis or stimulation of collagen production [Gauldie 1997].

Taken together, it appears that inhaled steroid treatment in COPD has only transient effects on functional outcome. The reasons for this are unknown, but need to be examined at the level of airway pathology. Therefore, we aim to investigate the clinical, functional as well as pathological outcome of short- and long-term treatment with inhaled steroids in patients with COPD. This will clarify whether short-term inhaled steroid treatment will suffice, or whether long-term treatment is still required to preserve the clinical, functional and/or pathological benefits. Since add-on therapy by long-acting ß2-agonists in combination with inhaled steroids has been demonstrated to be highly effective in asthma [Woolcock 1996, Pauwels 1998], we will also examine the short- and long-term benefits of such combination therapy in COPD. Short-term studies suggest that long-acting ß2-agonists may improve symptoms, quality of life, and exercise tolerance in COPD. However, this has not been formally addressed in longitudinal studies, nor in the presence of inhaled corticosteroids.

Hypothesis

We hypothesize that inhaled steroid treatment in patients with COPD leads to the following:

1. An initial improvement in symptoms, quality of life, and FEV_1, associated with a reduction in features of airways inflammation.
2. 6 Months of treatment will be sufficient to maintain these initial effects; continuation of therapy beyond 6 months will lead to an unfavourable balance between destruction and repair within the airway wall.
3. After the initial improvement, the increased annual decline in FEV_1 continues, which is associated with on-going airway remodelling and parenchymal destruction.
4. Airway hyperresponsiveness, elevated serum IgE, Th2 cytokine profile, and eosinophil infiltration are predictive of the initial benefits on symptoms, FEV_1, and airway inflammation.
5. Addition of a long-acting ß2-agonist to inhaled steroid treatment augments the benefit of therapy with regard to symptoms and quality of life, without changing the decline in FEV_1 or the pathological outcome.

Aim

The aim of the present study is to test the above hypotheses by comparing the clinical, functional and pathological benefits of:

- short-term (6 months) inhaled fluticasone followed by placebo (24 months)
- long-term (30 months) inhaled fluticasone
- long-term (30 months) salmeterol + inhaled fluticasone
- long-term (30 months) placebo

in a double blind, randomised, controlled trial in patients with COPD.

Outcome parameters

Primary

Inflammation

- localisation, numbers and profile of neutrophils, eosinophils, macrophages, and $CD8^+$ T cells in bronchial biopsy specimens

Secondary

Clinical

- symptoms, exacerbation rate, health-related quality of life, level and decline in FEV_1
- 6 minutes waling distance

Inflammation

- cellular and soluble markers in BAL and induced sputum
- profile of bronchial epithelial cells in bronchial biopsies
- markers of airway remodelling in bronchial biopsies

Pharmaco-economics

- cost-effectiveness
- cost-utility

Tertiary

Functional

- airway responsiveness (PC_{20}) to methacholine

Inflammation

- expression and localisation of pro- and anti-inflammatory cytokines or mediators in bronchial biopsies

Patient selection

Patient recruitment Groningen

Patients with COPD (n=135) will be selected from the practices of general practitioners and in the outpatient clinics of the Departments of Pulmonology from the University Hospital Groningen and the Leiden University Medical Center, and by advertisement through the media.

Participating general practitioners will be asked to select patients with COPD, who have not been treated with regular inhaled or oral corticosteroid during the last six months. In order to find these patients they will select patients between 45 and 75 years with a history of at least 10 pack years of smoking and at least one of the following symptoms: chronic cough, chronic sputum production, frequent exacerbations, or dyspnoea on exertion (see detailed inclusion and exclusion criteria, as listed below).

When selected the patient will be asked to cooperate in the study, and they will receive the patient information sheet. If in principle the patient is willing to cooperate, he or she will be referred to one of the study investigators. The study investigator will perform spirometry (FEV_1) in the investigational laboratory or at the home of the patient, and will provide detailed information to the patients about the study.

When the patient fulfils all the inclusion criteria and none of the exclusion criteria, informed consent must be signed by the patient and an appointment for visit one will be made.

Patient recruitment Leiden

In Leiden patients with COPD (n=135) will be recruited by advertisement through the media (appendix). Volunteers responding to this advertisement will first be screened by a telephone questionnaire taken by the investigators. When patients meet most of the inclusion criteria and if (in principle) they are willing to participate, they will be asked to visit the hospital to perform spirometry. Subsequently, when FEV_1 fulfills the inclusion criteria, the study investigator will provide information to the patient about the study, show a video demonstrating a bronchoscopy procedure and give the patient information form (appendix). One week later the investigator will telephone the patient and ask if he or she is willing to participate. If so, the study investigator will make an appointment for the first visit.

On this first visit the patient and investigator or his/her designee have to sign and date the general informed consent for participation in the study and an informed consent

concerning the three bronchoscopies (appendix).

After the first bronchoscopy the patient will be asked to complete a questionnaire about his or her experiences with this procedure. After having completed this questionnaire the patient will be asked specifically if he or she is still willing to participate in the study (appendix). If this is the case and the patient fulfills all the inclusion criteria and none of the exclusion criteria, the patient will be randomised and the next visit will be planned.

Inclusion criteria

1. Age: 45-75 years
2. $\geq$ 10 packyears of smoking
3. At least one of the following symptoms: chronic cough, chronic sputum production, frequent exacerbations, or dyspnoea on exertion
4. No course of oral corticosteroids during last 3 months, no maintenance treatment with inhaled or oral steroids during last 6 months
5. postbronchodilator value (after 400 μg of inhaled salbutamol) of FEV_1 below the 90% confidence interval (90% CI) of the predicted FEV_1, ánd postbronchodilator FEV_1/IVC ratio below the 90% CI of the predicted FEV_1/IVC ratio [Quanjer 1993]
6. postbronchodilator FEV_1 > 1.3 litre and > 20% of predicted value.
7. Written informed consent

Exclusion criteria

1. Prior or concomitant history of asthma
2. Alpha-1 antitrypsin deficiency (SZ, ZZ, zero phenotype)
3. Other active lung disease except for mild bronchiectasis; bronchiectasis should not be the main reason for chronic cough and/or sputum production with additional mild obstruction.
4. Contra-indications for elective bronchoscopy, such as O_2 saturation <90%, abnormal coagulability, anti-coagulant therapy which cannot be temporarily withheld for performance of bronchoscopy, history of pneumothorax, uncontrolled angina pectoris.
5. Other diseases likely to interfere with the purpose of the study.
6. Inability to keep diary and to understand written and oral instructions in Dutch.

Study design

In this prospective, longitudinal, double blind study COPD patients will be followed up for 2.5 years (*see figure*). The study begins as a 3 groups parallel design during 6 months, followed by a 4 groups parallel design during 24 months. The aim is to finish the study with 50 patients in each of the 4 parallel groups at 30 months.

Patients will be treated by the investigator-physician with inhaled corticosteroids (500 μg b.i.d. fluticasone by Discus[R]), or inhaled corticosteroids with long-acting $ß_2$-agonist (+ 50 μg b.i.d. salmeterol), or a matching placebo for the first 6 months. Thereafter, half of the corticosteroid group will continue and the other half will receive placebo for 2 years. The patients who will be treated with placebo or inhaled corticosteroids + salmeterol continue their treatment during the complete period of 2.5 years. Rescue treatment will be on demand usage of inhaled inpratropium bromide, which is a standard bronchodilator in COPD [Siafakas 1995].

Subjects who fulfil all inclusion criteria and none of the exclusion criteria (see patient selection) will be allocated to receive placebo, inhaled corticosteroids (equivalents of 500 μg b.i.d. = 1000 μg fluticasone) or inhaled corticosteroids + salmeterol at the beginning of the study using the minimization method. The use of minimization will provide treatment groups that are closely balanced for a number of variables (centre, gender, current smoker, FEV_1/IVC < 60%, PC_{20} methacholine < 2 mg/ml), which might influence the primary and secondary outcome variables [Altman 1995]. The first patient is given a treatment at random. For each subsequent patient we will determine which treatment will lead to better balance between the groups with respect to centre, gender, smoking, FEV_1/IVC and PC_{20}. The group with inhaled steroid will be randomly allocated to either the steroid group or the placebo group after 6 months of steroid treatment. Minimization will be performed using a computerized method [Pocock 1983].

Measurements

Measurements of symptoms, QOL, and spirometry (postbronchodilator) will be made every 3 months. In addition, exhaled nitric oxide (NO), bodyplethysmography and carbon-monoxide diffusion capacity will be measured at 0, 6 and 30 months. Bronchial biopsies, bronchoalveolar lavage, and sputum induction will also be performed at 0, 6 and 30 months. Peripheral blood eosinophils, IgE, and PC_{20} methacholine at 0, 6 and 30 months. In bronchial biopsies,

bronchoalveolar lavage and sputum special attention will be paid to the number of lymphocytes, neutrophils, eosinophils, macrophages, mast cells, and the state of activation of these cells and the epithelium.

Once during the study a spiral computed tomography (CT-scan) will be performed to quantify the presence of pulmonary emphysema in each patient [Gevenois 1996], and EDTA-anti-coagulated whole blood will be collected for DNA-isolation, for future studies on gene polymorphisms possibly associated with glucocorticoid responsiveness or other study outcomes.

The effects of treatment will be analysed by relating the observed changes in clinical and pathophysiological outcome, to those in cellular and histological outcome (see analysis).

Number of patients

In order to achieve a total number of n=200 (n=50 per treatment arm) at the end of the study, we will initially include 270 patients.

Withdrawal of patients

The patients are free to discontinue their participation in the study at any time and without prejudice to further treatment. The patient's participation in the study can be discontinued at any time at the discretion of the investigator. Justifiable reasons to discontinue a patient from the double blind treatment of the study include: the development of a serious adverse event, non-compliance of the patient, erroneous inclusion of the patient in the trial, acute exacerbations requiring oral steroid treatment (see below) for > 3 periods during the past 12 months or > 2 periods during the past 3 months, any treatment not allowed in the study, development of a concomitant disease that interferes with the interpretation of the study, withdrawal of consent. Patients who discontinue their participation in the study will be contacted by the investigator to obtain information about the reason for discontinuation. The reasons for study discontinuation will be reported on a study-termination form.

An effort will be made to collect as many data as possible on the outcome variables after withdrawal, so that the reasons for withdrawal can be analysed to check for bias.

Postponing visits in case of exacerbations

Any period requiring a short course of oral steroids (30 mg prednison for 6 days), given at the

discretion of the physician, will be labelled as an acute exacerbation. Study visits will have to be postponed if such oral course of steroids has been given < 8 weeks prior to the visit (12 weeks in case the visit will include a bronchoscopy. The next visit has to be scheduled at the next predefined time point according to the regular 3 monthly intervals.

Blinding

All study medication, active drugs and placebo will be of identical appearance throughout the study. The study inhalers will be labelled identically with centre code and subject number.

Treatment compliance

Patients are required to comply with the treatment regimens throughout participation in the study. Compliance will be checked by counting the doses on the Discus inhaler. A minimum of on average 70% of the treatment dose must have been taken in order to include a patient in the analysis.

Methods

Measurement of symptoms

Symptoms will be measured during the week before and the week after every visit with the Dutch version of the COPD Symptom Control Questionnaire (CSCQ). Additionally, the questionnaire will be administrated by the patients during the visit. Health related Quality of Life will be measured by generic and disease specific instruments.

Generic Health related QOL instruments

At the first visit, after six months and at the final visit patients will be asked to administer the Short Form 36 (SF36) [Ware 1992].

Disease specific instruments

At every visit patients will be asked to administer the St. George Respiratory Questionnaire (SGRQ) [Jones 1992].

Lung Function

Spirometry will be performed according to international guidelines [Quanjer 1993]. A weekly calibrated rolling-seal spirometer, or pneumotachograph (accuracy 50 ml) will be used throughout the study. First, 3 slow inspiratory vital capacity manoeuvres (IVC, use largest value) will be carried out. Second, maximally 5 forced expiratory vital capacity (FVC) manoeuvres will be performed to obtain at least 3 technically satisfactory expiratory flow-volume curves from which FVC does not deviate > 5% from the largest FVC. From these curves, we will use largest values of FVC, forced expiratory volume in 1 second (FEV_1), and instantaneous expiratory flows (MEF). Reference values will be obtained from Quanjer *et al.* [1993].

Reversibility of airways obstruction will be measured 20 min after administering 4 single puffs of 100 μg salbutamol per metered dose-inhaler connected to a spacer (Volumatic®), following the same procedures. The response will be expressed as change in FEV_1 as percentage of predicted value [Brand 1992].

At 0, 6 and 30 months total lung capacity (TLC), residual volume (RV), functional residual

capacity (FRC), airway resistance (Raw) and specific airway conductance (sGaw) will be measured using a constant volume bodyplethysmograph, according to a standardised method [Quanjer 1993]. Furthermore, the diffusion capacity (transfer factor) for carbon-monoxide (TL_{CO} and K_{CO}) will be measured using the single breathholding method [Quanjer 1993]. The single breath nitrogen wash-out test (slope of phase 3) will be added in Leiden only, as a simple parameter of small airway function with predictive properties for the development and course of COPD [Stanescu 1998].

Exercise capacity

At 0, 6 and 30 months the 6 minutes walking distance will be determined, in a flat indoor corridor, after 3 praticing attempts [Paggiaro 1998].

Airway hyperresponsiveness

Airway hyperresponsiveness will be determined using standardized methods [Sterk 1992, Demedts 1998]. This includes challenge tests with methacholine by so-called tidal breathing method. Serial doubling concentrations of methacholine-bromide (0.035-312 mg/ml) will be nebulized by a DeVilbiss 646 jet-nebulizer (filled with 3 ml, connected to an in- and expiratory valve box with an expiratory aerosol filter, operated with pressurized air) with monthly calibrated output of 130 mg/min. The aerosols will be inhaled by tidal breathing during 2 min at 5-min intervals through the mouth with the nose clipped. The response will be measured by FEV_1. After recording 3 reproducible (within 5% of largest) values of FEV_1 (to obtain mean baseline FEV1), the solvent will be used as first challenge. FEV_1 will be recorded at 30 and 90 s following each challenge, from which the lowest, technically satisfactory value will be used for further analysis. The first concentration of methacholine will be determined from recent guidelines [Demedts 1998]. The challenge will be discontinued if FEV_1 drops by > 20% from mean baseline value. Then the patient will receive 200 μg inhaled salbutamol per MDI, in order to reverse bronchoconstriction. The response of the challenges will be expressed as provocative concentration causing a 20% fall in FEV_1 (PC_{20}) by log-linear interpolation. Measurement of the fall in forced vital capacity (delta FVC) at the PC_{20} level is optional [Gibbons 1996].

Exhaled nitric oxide (NO)

Exhaled NO levels will be determined at 0, 6 and 30 months using the latest guidelines [Kharitonov 1997, ATS 1999] by a chemiluminescence analyzer (Sievers NOA 270B or Ecophysics CLD 700 AL) [Olin 1999]. The patients will be connected to a closed system with NO-free inspired air (pressurized air < 2 parts per billion: ppb, using a bag-system connected to a 3-way valve), to avoid contamination of the measurements with ambient NO. The patients will perform a slow vital expiratory capacity manoeuvre with a constant expiratory flow of 100 ml/sec against an expiratory resistance of 5 cm H_2O. Expiratory NO concentration is sampled continuously from the centre of the mouthpiece at a sample flow of 440 ml/min, and the average concentration will be determined during a period of 10 seconds. At each visit 3 successive recordings will be made at 30-s intervals, from which the mean values of exhaled NO will be used in the analysis.

Induced sputum

Sputum will be induced and processed according to a validated, and standardized technique [In 't Veen 1996], with some modifications. Prior to sputum induction, baseline FEV_1 will be recorded and, for safety reasons, 200 μg salbutamol will be administered through a metered dose inhaler (plus Volumatic®). Hypertonic sodium chloride aerosols (4.5 w/v %) will be generated at room temperature by a DeVilbiss Ultraneb 2000 ultrasonic nebulizer with a calibrated particle size (MMAD 4.5 μm) at maximal output (2.5 ml/min). The aerosols will be administered to the subjects through a 100 cm long tube with an internal diameter of 22 mm, and inhaled via the mouth through a two-way valve (No. 2700; Hans-Rudolph, Kansas City, MO, USA), with the nose clipped. Subsequently, the patients will inhale hypertonic saline aerosols during 3 periods of 5 min. After each inhalation, or as soon as the subjects experience cough, they are asked to blow their nose, to rinse their mouth and throat with water, and to expectorate sputum into a clean plastic container by coughing. Whenever the patients feels discomfort, and after completion of each period FEV_1 will be measured, and salbutamol will be administered if FEV_1 drops by > 20% from post-salbutamol baseline value.

Sputum processing

The volume of the induced sputum samples will be determined by weighing. The sample will then be mixed with an equal volume of 0.1% sputolysin (dithiotreitol, DTT, Calbiochem, USA).

To ensure complete homogenization, the samples are placed in a shaking water bath at 37ºC for 15 min, once interrupted by gently mixing the sample. The homogenized sputum will be centrifuged (350 x g) for 10 mins at 4°C. The cell-free supernatant will be collected and stored in aliquots at -80°C pending analysis of soluble mediators. The cell pellet will then be resuspended in phosphate-buffered saline (PBS) containing 1 % (w/v) human serum albumin (HSA), pH 7.4, to a final volume of 2-5 ml, followed by filtration through a nylon gauze (pore-size approximately 48 μm, Thompson, Ontario) to remove clumps (filter method to be standardized to a uniform method in Groningen and Leiden). Cell viability and total cell counts are performed by Trypan blue exclusion and using a hemacytometer (use of automated cell count using e.g. Coulter Counter to be discussed). Subsequently the sample will be diluted with PBS (with 2 % w/v HSA, pH 7.4) to a final concentration of 0.3-0.4 x 10^{6} cells/ml which is used for preparation of the cytocentrifuge slides (Leiden: 1500 rpm, 3 mins, 50 μl/slide, Shandon 3, Life Sciences International, Veldhoven, The Netherlands; Groningen: 450 rpm, 6 mins, 100 μl/slide; centrifugation speed and equipment to be standardized to a uniform method in Groningen and Leiden). Two slides will be used for Diff-Quik staining to obtain cell differentials, 6-8 slides will be dried and stored at -80°C pending immunohistochemistry analysis.

Differential cell counts of eosinophils, neutrophils, lymphocytes, macro-phages, epithelial and squamous cells are performed on Diff-Quik (choice of staining to be discussed) stained cytospins by a qualified cytopathologist. To correct for the variable salivary contamination, differential leucocyte and cylindric epithelial cell counts will be expressed as a percentage of 500 nucleated cells excluding squamous cells. For each sample, differential cell counts will be performed twice by the same observer, and the mean data will be used in the analysis. A sputum sample will be considered adequate when the percentage squamous cells is less than 80%. To ensure a blind analysis of the sputum samples, all cytocentrifuge slides are coded before analysis by an investigator who is not involved in the counting.

Sputum supernatant will be used to measure soluble mediators (see elsewhere in this section).

Bronchoscopy

Bronchoscopy will be performed using established guidelines in general and those developed for asthma in particular [NHLBI 1985, Harrison 1993, Jarjour 1998]. Smokers are requested

not to smoke their first cigarette on the day of the bronchoscopy before all procedures for investigation are completed. Patients are not allowed to drink or eat food from midnight onwards, except for taking their medication. On arrival for bronchoscopy they will receive 400 μg salbutamol per Volumatic[R] spacer, 20 mg oral codeine phosphate and 0.5 mg of atropine s.c. [Harrison 1993]. Fifteen minutes later 2 ml of lidocain 1-4% will be instilled in the mouth 3-5 times, on the vocal cords and into the trachea to inhibit coughing. The total lidocain dose must not exceed 3 mg/kg [Ghio 1998]. A flexible bronchoscope will be introduced and bronchoalveolar lavage will be performed into the right middle lobe. 4 Times 50 ml PBS at 30ºC will be instilled and after 10 sec of dwelling, it will be removed by gentile suction at 20 cm water pressure or less. Thereafter, 3 times 50 ml of saline will be instilled with dwelling times of 10 sec and each portion of 50 ml of saline will be collected in a tube before the next 50 mls are instilled. All collected BAL will be immediately stored on ice and transported to the laboratory within 15 min after collection. Separate analysis of the first aliquot will be considered.

Bronchial biopsies will be taken from subsegmental carinae in the right or left lower lobe using cup forceps. When possible at least 6 biopsies will be taken (Aleva 1998). Four biopsies will be fixed in 4% neutral buffered formalin, processed and embedded in paraffin. The remaining biopsies will be embedded in Tissue Tek mounting medium, snap frozen in liquid isopentane and finally stored at -80°C.

Processing of BAL fluid

BAL processing will be performed analogous to the methods used for sputum processing, with the major exception that no DTT/Sputolysin will be used for homogenization.

Immediately upon collection, the BAL fluid will be placed on ice and centrifuged (350 x g) for 10 min at 4^oC. The cell-free supernatant will be collected and stored in aliquots at -80^oC pending analysis of soluble mediators.

The cell pellet will then be resuspended in phosphate-buffered saline (PBS) containing 1 % (w/v) human serum albumin (HSA), pH 7.4, to a final volume of appr. 1 ml, followed by filtration through a nylon gauze (pore-size approximately 48 μm, Thompson, Ontario) to remove clumps (filter method to be standardized to a uniform method in Groningen and Leiden). Cell viability and total cell counts are performed by Trypan blue exclusion and using a hemacytometer (use of automated cell count using e.g. Coulter Counter to be discussed). Cytospin preparation and

analysis will be performed as described in the Sputum Processing paragraph of this section.

Immunocytochemistry of sputum and BAL cells

Immunocytochemical staining for cytokines and SLPI as described in the biopsy paragraph will be performed on cytospin preparations of BAL and sputum. Staining will be performed on formalin-fixed, saponin-permeabilized preparations as described [Grunberg, 1997; details to be discussed between Leiden and Groningen].

Assessment of mediator concentration in sputum and BAL

For all mediators to be assessed in sputum, initial experiments will be performed to optimize these assays in this complex biological fluid [Grünberg 1997]. These experiments include assessment of the recovery of the mediator and the effect of the reducing agent dithiotreitol on the immunoassay for that specific mediator.

Cytokine and SLPI levels. Cytokine levels (IL-1, TNF-α, IL-6, IL-8, IL-4, IFN-γ, IL-10 and TGF-β) will be assessed using commercially available immunoassays (Elisa). Levels of SLPI (also known as ALP) will be determined using an Elisa developed in the laboratory of Leiden [Kramps 1984, Wingens 1998]. IL-5 will be quantified with an Elisa procedure developed in Groningen.

Additional measurements. Levels of albumin, eosinophil cationic protein (ECP), neutrophil elastase will be determined as described [*e.g.* Grünberg 1997]. BAL GSH levels will be determined using a spectrophotometric method [Rahman 1998].

Histology

Frozen tissue: Four μm thick frozen sections will be cut and stored at -20°C until use. In frozen sections the haematoxylin-eosin (H-E) staining will be used for judging the biopsy quality. These HE slides allow a first impression of the degree of inflammation and give an indication of the types of inflammatory cells. The first screening of biopsies is performed on haematoxylin and eosin stained frozen sections.

Paraffin-embedded tissue: From paraffin-embedded tissues 3 μm thick sections are cut. H-E staining will be used for evaluation of overall bronchial architecture, epithelial integrity, and extent of the inflammatory infiltrate. Giemsa stain will be used for further indication of cell populations (eosinophils, mast cells) and PAS diastase stain for basal membranes.

Immunohistochemistry

Frozen tissue: Immunohistochemistry is performed on 4 μm frozen sections using two and three step immunoperoxidase protocols. The two-step method is done according to standard operation protocols which are available on request. Briefly, the sections are air-dried overnight and fixed in fresh acetone for 10 mins at room temperature. Sections are washed in phosphate-buffered saline (PBS) after each incubation. Sections are pre-incubated for 30 mins with 10% normal rabbit or goat serum or 1% bovine serum albumin (BSA) to avoid non-specific immunoreactivities. The preincubation fluid is decanted and next, without washing, the primary antibody is incubated for 60 minutes. After washing, the slides are incubated with appropriate secondary antibodies conjugated to horseradish peroxidase. To prevent non-specific staining, 1% normal serum or BSA is added to the second step reagent. The peroxidase label is detected with 3-amino-9-ethylcarbazol or 3,3'-diaminobenzidin together with H_2O_2. Sections are counter stained with haematoxylin. Negative controls include omission of the primary antibody and application of immunoglobulin-subclass-matched, animal source matched, non-relevant antibodies (if available).

Two different three-step immunoperoxidase protocols are performed to enhance the signal of weakly staining antibodies. The first three-step protocol includes incubation with the second step reagent biotin-labelled secondary antibody followed by incubation with horseradish peroxidase-labelled streptavidin (SBA). The second three-step protocol consists of incubation with horseradish peroxidase-labelled rabbit anti-mouse immunoglobulin antiserum as the second step reagent, followed by horseradish peroxidase-labelled goat or swine anti-rabbit immunoglobulin antiserum as the third step reagent. Other incubation steps are performed as described in the two step immunoperoxidase protocol.

Paraffin tissue

Immunohistochemistry is performed on 3 μm formalin fixed, paraffin embedded lung tissue sections using two or three step immunoperoxidase protocols as described in standard operation protocols. Both protocols are mainly as described for frozen sections. In short, to improve the staining quality of some of the antibodies, antigen retrieval is performed by either proteolytic treatment or by heating in either EDTA (pH 8.0) or citrate buffer (pH 6.0).

Endogenous peroxidase activity is blocked by incubation of the sections for 30 minutes in

H_2O_2. Subsequent to pretreatment (if necessary), sections are washed and pre-incubated for 30 minutes with 10% normal serum or 1% BSA to avoid non-specific immunoreactivities. Sections are then incubated with the primary antibody overnight. The remainder of the protocols is as described for frozen sections.

Biopsy parameters to be studied

In the following, an overview is given of the possible assessments in biopsies. The number of immunostainings (different antigens) to be performed is determined by the number of available biopsies and their quality.

<u>Lymphocytes:</u>

-T cell subsets (CD3, CD4, CD8) and NK cells (CD56)

-Determination of activation of T-cells (CD4, CD8) by staining for T-cell markers and IL2R/CD25

<u>Mast cells:</u>

-number and localization of mast cells (Giemsa; tryptase)

<u>Neutrophils</u>

-localization and number of neutrophils (neutrophil elastase)

<u>Eosinophils:</u>

-number and localization of eosinophils and activated (EG2+) eosinophils

<u>Macrophages:</u>

-CD68

<u>Epithelium:</u>

-qualitative evaluation (a.o. “intactness”)

-morphological changes, incl. metaplasia

-adhesion molecules: ICAM-1, VCAM-1, E-cadherin, EpCam

Vessels:

-determination of ICAM-1, VCAM-1, E-selectin and P-selectin on vascular endothelium, as these molecules are highly sensitive for regulatory mechanisms in inflammation (in double-staining with CD31).

Cytokines

IL1, TNF-alpha, IL6, IL8, IL4, IL5, IFN-gamma, IL10, TGF-beta

Other:

SLPI

Quantitative assessments in bronchial biopsies

All biopsy specimens will be coded and sections will be examined by a single investigator in a blinded fashion, by means of a semi-automated image analysis system using a 200x magnification. Images of areas with well preserved tissue structure and without bronchus associated lymphoid tissue will serve as basis for further computer-aided analysis. For subepithelial analysis, the widest possible zone of maximal 125 μm deep beneath the epithelial basement membrane will be delineated excluding damaged tissue, mucus-secreting glands, and airway smooth muscle (Sont 1996, 1997; Grashoff, 1997; Ten Hacken 1997, 1998). For epithelial analysis, the epithelium will be regarded using the same criteria (Grashoff, 1997). The number of positively staining nucleated inflammatory cells will be counted by a validated full automated procedure (Sont 1998) within the largest possible area per biopsy section (minimally 20,000 μm^2), and expressed as the number of cells / 0.1 mm^2 (subepithelial) or per 0.1 mm^2 and per mm basement membrane (epithelial). Expression of cytokines and SLPI in the airway epithelium and lamina propria will be semi-quantitatively scored (Sont 1997; de Boer 1998) and quantitatively assessed by densitometric analysis using a digital image analysis system.

Computed tomography

At the start of the study the degree of pulmonary emphysema will be quantified in each COPD patient by CT-scan [Gevenois 1996]. Single slices will be obtained from lung apex to basis (120 kVp, 200 mA, scan time 1 s per slice, 1 mm thickness, 1 cm interval). The acquired

images will be analysed to calculate mean lung density expressed in Hounsfield Units (HU) [Cheung 1997].

Data analyses

Determination of the sample size

The determination of the sample size is based on detectable differences in the primary outcome variables, the mean inflammatory cell counts in bronchial biopsies between the different treatment groups. Under the assumption that the distributions of (logtransformed) cell counts is approximately normal and the standard deviation in the different treatment groups is approximately equal, the sample size follows:

A sample size of 8-25 patients is needed to detect at least one doubling difference in mean cell number per 0.1mm^2 for a particular inflammatory cell to detect changes within a group (alpha=0.05, power=0.80). A sample size of 13-48 subjects per group is required to detect at least 1 doubling difference in cell number between parallel groups [Sont 1997].

Based on an estimated standard deviation of repeated cell counts in bronchial biopsies of 2.34 fold change, a sample size of 50 per study arm allows us to detect at least a 1.62 fold different increase in number of inflammatory cells/0.1 mm^2 between two groups.

Statistical methods

Quality of data: The quality of the data entry will be checked in a random sample of 10% of the cases.

Safety: During the data entry process, all Case Report Forms will be reviewed for possible adverse events (AE). Adverse events are defined as: any unfavourable, unintended event (signs, symptoms, changes in laboratory data) temporarily associated with the administration of the study drug whether or not considered drug related. Examples are signs, symptoms or disorders reflecting adverse event found anywhere in the Case Report Form as notes or comments. All data regarding serious adverse events will be entered into a safety database and analysed at the department of Epidemiology & Statistics of the University of Groningen, 12 months after the start of the study and again after 24 months. The safety variables that will be analysed at those time points are: spontaneously reported adverse events, extended adverse events, question on diagnosis of conditions associated with glucocorticosteroid treatment (hypertension, bone fractures, subcapsular posterior cataract, myopathy and diabetes, and skin bruises on both forearms).

Statistical analyses

Prior to breaking the treatment code at the end of the study, all decisions on the evaluability of data from each individual patient for the statistical analyses are made and documented. The effects of treatment will be analysed according to treatment group. Primary analyses will be comparing changes in cell counts per treatment group in time. Secondary analyses will compare changes in clinical outcome variables such as lung function and respiratory symptoms with changes in cell counts.

Linear mixed effects models will be used to estimate the effects of treatment on changes in primary outcome variables (cell counts) and the secondary outcome variables (such as lung function, bronchial hyperresponsiveness, number of blood eosinophil count). Graphical visual inspection will be used to determine which model has the best fit (linear/non-linear model). Descriptive statistics will be presented by visit. Confidence intervals will be computed using a 95% confidence interval level and p-values <0.05 are considered to be statistically significant.

In addition, the baseline values of lung function, bronchial biopsy, bronchoalveolar lavage and sputum induction will be evaluated as possible explanatory variables for the outcome. Smoking habits will be evaluated as the duration of smoking (years), average daily consumption prior to the study, average daily consumption during the periods between visits, previous cigarette consumption expressed as packyears.

Time points will be calculated as time elapsed since baseline visit.

References

1. Abbinante-Nissen JM, Simpson LG, Leikauf GD. Corticosteroids increase secretory leukocyte protease inhibitor transcript levels in airway epithelial cells. Am J Physiol 1995;12:L601-L606.
2. Aleva RM, Kraan J, Smith M, Ten Hacken NHT, Postma DS, Timens W. Techniques in human airway inflammation - Quantity and morphology of bronchial biopsy specimens taken by forceps of three sizes. Chest 1998;113:182-185.
3. Altman DC. Practical statistics for medical research. London: Chapman & Hall, 1995: 443-445.
4. American Thoracic Society. Recommendations for a standardized procedure for the on-line and off-line measurement of exhaled lower respiratory nitric oxide and nasal nitric oxide in adults and children. Am J Respir Crit Care Med 1998;in press.
5. Brand PLP, Quanjer PH, Postma DS, Kerstjens HAM, Koeter GH, Dekhuijzen R, Sluiter HJ, and the Dutch CNSLD study group. Interpretation of bronchodilator response in patients with obstructive airways disease. Thorax 1992;47:429-436.
6. Chanez P, Vignola AM, O'Shaughnessy T, Enander I, Li D, Jeffery PK, Bousquet J. Corticosteroid reversibility in COPD is related to features of asthma. Am J Respir Crit Care Med 1997;155:1529-1534.
7. Cheung D, Schot R, Zwinderman AH, Zagers H, Dijkman JH, Sterk PJ. Relationship between loss in parenchymal elastic recoil pressure and maximal airway narrowing in subjects with alpha-1-antitrypsin deficiency. Am J Respir Crit Care Med 1997;155:135-140.
8. De Boer WI, van Schadewijck WAAM, Sont JK, Sharma HS, Stolk J, Hiemstra PS, van Krieken JHJM. Transforming growth factor ß1 and recruitment of macrophages and mast cells in airways in chronic obstructive pulmonary disease. Am J Respir Crit Care Med 1998, in press.
9. Demedts M, Decramer M. Longfunctie onderzoek. Garant, Leuven/Apeldoorn, 1998:p195-p 212.
10. Dompeling E, van Schayck CP, Molema J, Folgering H, van Grunsven PM, van Weel C. Inhaled becolmethasone improves the course of asthma and COPD. Eur Respir J 1992;5:945-952.
11. Gauldie J, Sime PJ, Tremblay GM, Torry D, Jordana M. Tissue remodeling and fibroblast heterogeneity in asthma and other chronic airways inflammatory diseases. In : Inhaled glucocorticoids in asthma. Mechanisms and clinical actions. Ed. R.P. Schleimer, W.W. Busse, P.M. O'Byrne. Marcel Dekker Inc., New York, 1997:p151-p166.
12. Gevenois PA, de Vuyst P, de Maertelaer V, Zanen J, Jacobovitz D, Cosio MG, Yernault J-C. Comparison of computed density and microscopic morphometry in pulmonary emphysema. Am J Respir Crit Care Med 1996;154:187-192.
13. Ghio AJ, Bassett M, Chall A, Levin D, Bromberg PhA. Bronchoscopy in healthy volunteers. J Bronchol 1998;5:185-194.
14. Gibbons WJ, Sharma A, Lougheed D, Macklem PT. Detection of excessive bronchoconstriction in asthma. Am J Respir Crit Care Med 1996;153:582-589.
15. Grashoff WFM, Sont JK, Sterk PJ, Hiemstra PS, de Boer WI, Stolk J, van Krieken JHJM. Chronic obstructive pulmonary disease. Role of bronchiolar mast cells and macrophages. Am J Pathol 1997;151:1785-1790.
16. Grünberg K, Smits HH, Timmers MC, De Klerk EPA, Dolhain RJEM, Dick EC, ra PS, Sterk PJ. Experimental rhinovirus 16 infection. Effects on cell differentials and soluble markers in sputum in asthmatic subjects. Am J Respir Crit Care Med 1997;156:609-

16.
17. In 't Veen JCCM, de Gouw HWFM, Smits HH, Sont JK, Hiemstra PS, Sterk PJ, Bel EH. Repeatability of cellular and soluble markers of inflammation in induced sputum from patients with asthma. Eur Respir J 1996;9:2441-1447.
18. Jones PW, Quirk FH, Baveystock CM, Littlejohns P. A self-complete measure of health status for chronic airflow limitation. Am Rev Respir Dis 1992;145:1321-1327.
19. Kharitonov S, Alving K, Barnes PJ. Exhaled and nasal nitric oxide measurements: recommendations. Eur Respir J 1997;10:1683-1693.
20. Klech H, Pohl W. Technical recommendations and guidelines for bronchoalveolar lavage (BAL). Eur Respir J 1989;2:561-585.
21. Kramps JA, Franken C, Dijkman JH. ELISA for quantitative measurement of low-molecular-weight bronchial protease inhibitor in serum. Am Rev Respir Dis 1984;129:959-963.
22. Jarjour NN, Peters SP, Djukanovic R, Calhoun WJ. Investigative use of bronchoscopy in asthma. Am J Respir Crit Care Med 1998;157:692-697.
23. Llewellyn-Jones CG, Harris TAJ, Stockley RA, Harris TA. Effect of fluticasone proprionate on sputum of patients with chronic bronchitis and emphysema. Am J Respir Crit Care Med 1996;153:616-621.
24. NHLBI. Workshop Summaries. Summary and recommendations of a workshop on the investigative use of fiberoptic bronchoscopy and bronchoalveolar lavage in athmatics. Am Rev Respir Dis 1985;132:180-182.
25. Olin AC, de Gouw HWFM, Ljungkvist G, Schot R, Sterk PJ, Torén K. Flow dependency of exhaled nitric oxide (NO) in asthmatics and controls: comparison of two analysers. Am J Respir Crit Care Med 1999;abstract submitted.
26. O'Shaughnessy TC, Ansari TW, Barnes NC, Jeffery PK. Inflammation in bronchial biopsies of subjects with chronic bronchitis: inverse relationship of CD8+ T lymphocytes with FEV_1. Am J Respir Crit Care Med 1997;155:852-857.
27. Paggiaro PL, Dahle R, Bakran I, Frith L, Hollingworth K, Efhtimiou J. Multicentre randomised placebo-controlled trial of inhaled fluticasone-proprionate in patients with chronic obstructive pulmonary disease. Lancet 1998;351:773-780.
28. Pauwels RA, Lofdahl C-G, Postma DS, Tattersfield AE, O'Byrne PM, Barnes PJ, Ullman A. Effect of inhaled formoterol and budesonide on exacerbations of asthma. N Engl J Med 1997;337:1405-1411.
29. Pocock SJ. Clinical trials, a practical approach. Chichester: John Wiley & Sons, 1983:84-87.
30. Postma DS, Peters I, Steenhuis EJ, Sluiter HJ. Moderately severe chronic airflow obstruction. Can corticosteroids slow down obstruction? Eur Respir J 1988;1:22-26.
31. Postma DS. Inhaled therapy in COPD: what are the benefits? Respir Med 1991;85:447-449.
32. Postma DS, Siafakas NM. Management of chronic obstructive pulmonary disease. Eur Respir Monograph 1998;3:1-302.
33. Quanjer PhH, Tammeling GJ, Cotes JE, Pedersen EF, Peslin R, Yernault J-C. Lung volumes and forced expiratory flows. Eur Respir J 1993;6(suppl 16):5-100.
34. Rahman I, Bel A, Mulier B, Donaldson K, MacNee W. Differential regulation of glutathione by oxidants and dexamethasone in alveolar epithelial cells. Am J Physiol 1998;275:L80-L86.
35. Renkema TEJ, Schouten JP, Koëter GH, Postma DS. Effects of long-term treatment with corticosteroids in chronic obstructive pulmonary disease. Chest 1996;109:1156-1162.

36. Riess A, Wiggs B, Verbrugt L, Wright JL, Hogg JC, Pare PD. Morphologic determinants of airway responsiveness in chronic smokers. Am J Respir Crit Care Med 1996;154:1444-1449.
37. Saetta M, Finkelstein R, Cosio MG. Morphological and cellular basis for airflow limitation in smokers. Eur Respir J 1994;7:1505-1515.
38. Saetta M, di Stefano A, Turato G, Facchini FM, Corbino L, Mapp CE, Maestrelli P, Ciaccia A, Fabbri LM. CD8+ T-lymphocytes in peripheral airways of smokers with chronic obstructive pulmonary disease. Am J Respir Crit Care Med 1998;157:822-826.
39. Scholten JHG, van Weel C. Functional Status assessment in Family Practice. The Darmouth COOP Functional Health Assessment Charts/WONCA. Lelystad MEDItekst 1992. Medt 1992;......
40. Siafakas NM, Vermeire P, Pride NB, Paoletti P, Gibson J, Howard P, Yernault Jc, Decramer M, Higenbottam T, Postma DS, Rees J. Optimal assessment and management of chronic obstructive pulmonary disease. Eur Respir J 1995;8: 1398-1420.
41. Sont JK, Willems LN, Evertse CE, Hooijer R, Sterk PJ, van KJ. Repeatability of measures of inflammatory cell number in bronchial biopsies in atopic asthma. Eur Respir J 1997;10:2602-2608.
42. Sont JK, van Krieken JHJM, van Klink HCJ, Roldaan AC, Apap CR, Willems LNA, Sterk PJ. 1997. Enhanced expression of neutral endopeptidase (NEP) in airway epithelium in biopsies from steroid- versus nonsteroid-treated patients with atopic asthma. Am J Respir Cell Mol Biol 16:549-556.
43. Sont JK, Grünberg K, Sterk PJ, van Krieken JHJM. Automated assessment of inflammatory cell counts in bronchial biopsy specimens: repeatability and agreement with interactive point counting. Eur Respir J 1998;12(suppl.28):195s.
44. Sterk PJ, Fabbri LM, Quanjer PhH, Cockcroft DW, O'Byrne PM, Anderson SD, Juniper EF, Malo J-L. Airway responsiveness. Standardized challenge testing with pharmacological, physical and sensitizing stimuli in adults. Eur Respir J 1993;6(suppl16):53-83.
45. Stanescu D, Sanna A, Veriter C, Robert A. Identification of smokers susceptible to development of chronic airflow limitation. A 13-years follow-up. Chest 1998;114:416-425.
46. Tashkin DP, Altose MD, Connett JE, Kanner RE, Lee WW, Wise RA, for the Lung Health Study Research Group. Methacholine reactivity predicts changes in lung function over time in smokers with early chronic obstructive pulmonary disease. Am J Respir Crit Care Med 1996;153:1802-1811.
47. Ten Hacken NHT, Aleva RM, Rutgers B, Kraan J, Postma DS, Timens W. Differences in plastic embedded and snap-frozen sections for CD3, CD4 and CD8 immunostaining of bronchial biopsies. Modern Pathol 1997;10:1043-1046.
48. Ten Hacken NHT, Aleva RM, Oosterhoff Y, Smith M, Kraan J, Postma DS, et al. Submucosa 1.0 x 0.1 mm in size is sufficient to count inflammatory cell numbers in human airway biopsies. Modern Pathol 1998;11:292-294.
49. Ten Hacken NHT, Postma DS, Bosma F, Drok G, Kraan J, Timens W. Vascular adhesion molecules in nocturnal asthma: a possible role for VCAM-1 in ongoing airway wall inflammation. Clin Exp Allergy 1998;in press.
50. Thurlbeck WM. Pathophysiology of chronic obstructive pulmonary disease. Clin Chest Med 1990;11:389-403.
51. Timens W, Coers W, van Straaten JFM, Postma DS. Extracellular matrix and

inflammation: a role for fibroblast-mediated tissue repair in the pathogenesis of emphysema? Eur Respir Rev 1997;7:119-123.
52. Villar MTA, Dow L, Coggon D, Lampe FC, Holgate ST. The influence of increased bronchial responsiveness, atopy, and serum IgE on decline in FEV1. A longitudinal study in the elderly. Am J Respir Crit Care Med 1995;151:656-662.
53. Ware JE, Sherbourne CD. The MOS-36-item Short-form Health Status Survey (SF-36):1. Conceptual framework and item selection. Med Care 1992;30:473-483.
54. Wingens M, van Bergen BH, Hiemstra PS, et al. Induction of SLPI (ALP/HUSI-I) in epidermal keratinocytes. J Invest Derm 1998; [In Press].
55. Woolcock AJ, Lundback B, Ringdal N, Jacques LA. Comparison of addition of salmeterol to inhaled steroids with doubling of the dose of inhaled steroids. Am J Respir Crit Care Med 1996;153:1481-1488.

Advertentie Leiden:

OPROEP WETENSCHAPPELIJK ONDERZOEK

Heeft u last van hoesten, slijm opgeven of kortademigheid?

Afdeling Longziekten
Huisartsgeneeskunde

- Heeft u tenminste 10 jaar gerookt?
- Bent u tussen de 45 en 75 jaar oud?
- Bent u eventueel al bekend met chronische bronchitis en/of longemfyseem?
- En wilt u tevens deelnemen aan wetenschappelijk onderzoek ter verbetering van de behandeling van CARA/COPD?

Neemt u dan contact op met de artsen: J. Stroband of T. Lapperre, Leids Universitair Medisch Centrum, afd. Longziekten, tel: 071-5263700 (tijdens kantooruren).
Wij bieden u medisch onderzoek, deskundige begeleiding en onkostenvergoeding.

PROEFPERSONEN INFORMATIE

TEN BEHOEVE VAN WETENSCHAPPELIJK

ONDERZOEK NAAR:

Verbetering van de behandeling
bij patiënten met COPD

G roningen
L eiden
U niversities
C orticosteroids in
O bstructive
L ung
D isease

Geachte mevrouw, mijnheer,

In aansluiting op uw vrijwillige reactie op onze advertentie en uw bezoek aan het ziekenhuis ontvangt u hierbij de schriftelijke informatie over het wetenschappelijk onderzoek naar de behandeling van patiënten met chronische obstructieve longziekte (COPD). De behandeling zal gebeuren met inhalatiesteroïden, een ontstekingsremmer, al dan niet in combinatie met een langwerkende luchtwegverwijder. Het is belangrijk dat u deze informatie goed leest voordat u besluit of u aan het onderzoek wilt meedoen. Denk er thuis rustig over na. Als u vragen heeft, bespreek ze dan vooral met uw behandelend arts of met een van ons.

Achtergrond van het onderzoek

Vroeger werd voor chronische longziekten de term CARA gebruikt. Hiertoe behoorde astma, emfyseem en chronische bronchitis. De laatste jaren bleek echter steeds duidelijker een verschil te bestaan tussen astma aan de ene kant en chronische bronchitis en emfyseem aan de andere kant. Astma wordt voornamelijk gekenmerkt door een wisselende luchtwegvernauwing, terwijl bij emfyseem en chronische bronchitis meestal een langzaam in ernst toenemende continue luchtwegvernauwing bestaat. Tegenwoordig wordt daarom voor de laatste twee ziekten de term COPD gebruikt. De belangrijkste symptomen van COPD zijn: kortademigheid bij inspanning, hoesten, slijm opgeven en prikkelbaarheid van de luchtwegen voor bepaalde prikkels als rook, mist en kou. Op den duur leidt deze aandoening bij een deel van de patiënten tot ernstige invaliditeit. COPD is momenteel de vijfde belangrijkste doodsoorzaak in de wereld en het aandeel neemt nog toe.

De enige behandeling die de snelle achteruitgang in longfunctie bij COPD patiënten kan remmen is stoppen met roken. Daarnaast kunnen bepaalde luchtwegverwijders (vaak atrovent) of inhalatiesteroïden worden gegeven en tijdens perioden van verergering van de klachten (exacerbaties) antibiotica- en/of prednison- kuren. Geadviseerd wordt een jaarlijkse griepvaccinatie te nemen, op een goed gewicht te blijven en voldoende lichaamsbeweging te hebben. Recente onderzoeken lieten zien dat inhalatiesteroïden in de eerste 3-6 maanden mogelijk de longfunctie constant houden of zelfs verbeteren, echter daarna leek er geen groot effect op de longfunctie meer te zijn. Ander onderzoek heeft aangetoond dat een langwerkende luchtwegverwijder als salmeterol (serevent) de kortademigheid kan verminderen. Het is niet duidelijk of langdurige behandeling met dit medicijn de achteruitgang van de longfunctie voorkomt.

Doel van het onderzoek

Het doel van het onderzoek waarvoor wij uw medewerking vragen is om na te gaan of het voor het succes van de behandeling uitmaakt of inhalatiesteroïden voor korte (6 maanden) of lange (2.5 jaar)

duur worden gegeven. Daarnaast onderzoeken we het effect van een langwerkende luchtwegverwijder in combinatie met inhalatiesteroïden.

De opzet van het onderzoek

Via advertenties zoeken wij in Leiden 130 patiënten met COPD, die onder andere tussen de 45 en 75 jaar oud zijn, tenminste 10 jaar hebben gerookt en last hebben van benauwdheid, hoesten of slijm opgeven. Deze patiënten worden willekeurig verdeeld over één van de volgende 4 behandelgroepen:

- 6 maanden inhalatiesteroïden (fluticason), gevolgd door 2 jaar placebo*,
- 2.5 jaar inhalatiesteroïden (fluticason),
- 2.5 jaar inhalatiesteroïden in combinatie met een langwerkende luchtwegverwijder (fluticason en salmeterol) of
- 2.5 jaar placebo*

  * de inhalatiesteroïden worden vergeleken met een middel (=placebo), dat er wel hetzelfde uitziet, maar dat geen steroïden bevat, een “nep-medicijn”

Wat wordt er van u gevraagd?

Op 0, 6 en 30 maanden wordt u uitgebreid onderzocht op vier aparte dagen:

- op dag 1 krijgt u gedetailleerde informatie over het onderzoek en laten wij u een video over een bronchoscopie zien. Als u hierna besluit om deel te nemen aan het onderzoek vragen wij u of u een algemeen toestemmingsformulier wilt ondertekenen. Aansluitend wordt een hartfilmpje en een longfoto gemaakt, lichamelijk onderzoek gedaan, bloed afgenomen en longfunctie onderzoek gedaan
- op dag 2 wordt u gevraagd vragenlijsten in te vullen en slijm (= sputum) op te geven dat we kunnen onderzoeken
- op dag 3 wordt een blaastest verricht waarbij NO in de uitademingslucht wordt gemeten en er wordt een 6 minuten looptest gedaan
- op dag 4 wordt in de longen gekeken tijdens een zogeheten 'bronchoscopie'.

Zoals afgesproken wordt u gevraagd om na de eerste bronchoscopie (op dag 4 van het eerste bezoek) een vragenlijst over deze ingreep in te vullen en nogmaals te beslissen over verdere deelname aan dit project. Deze vragenlijst vormt voor u de basis om verder te participeren in dit onderzoek. U kunt rustig na denken over verdere deelname aan de studie. Wij zullen contact met u opnemen en dan met u bespreken welke beslissing u heeft genomen. Indien u deel wilt nemen aan het vervolg van het onderzoek verzoeken wij u de vraag hierover aan het einde van de vragenlijst te beantwoorden en de vragenlijst naar ons te retourneren.

Gedurende het onderzoek bezoekt u elke drie maanden het ziekenhuis om vragenlijsten in te vullen en een longfunctie onderzoek te doen. In totaal duurt het onderzoek 2,5 jaar, bestaande uit 11 bezoeken aan het Leids Universtair Medisch centrum (LUMC).

Informatie over specifieke onderzoeken

De vragenlijsten hebben betrekking op longklachten, andere ziekten, medicijn gebruik en algemene informatie. Het invullen duurt ongeveer ¾ uur.

Een bronchoscopie is een vaak toegepast kijk-onderzoek van de grote luchtwegen. Na een plaatselijke verdoving met een druppel- en inhalatievloeistof wordt een dun kijk-slangetje via de neus of mond in de grote luchtwegen gebracht. De arts inspecteert vervolgens de luchtwegen en neemt kleine slijmvlieshapjes van ongeveer 1x1 mm groot (zgn. biopten). Het nemen van deze

biopten is niet pijnlijk. Wel kan deze procedure soms tot kortademigheid leiden. Daarom wordt van tevoren een luchtwegverwijder gegeven (salbutamol) en tijdens het onderzoek zuurstof via een neusslangetje. Het is mogelijk dat u na afloop van het onderzoek kortdurend een sliertje bloed ophoest. U hoeft zich hier geen zorgen over te maken. Met voor- en nazorg duurt de bronchoscopie ca. 1½ uur.

'Sputum-inductie' is een onderzoek waarbij slijm (sputum) wordt verkregen uit de luchtwegen. Drie keer wordt u verzocht gedurende 5 minuten een vernevelde oplossing met water en zout in te ademen. Na elke inhalatieperiode wordt u gevraagd slijm op te geven in een bakje. Omdat u hier misschien benauwd van kunt worden, geven we van tevoren een luchtwegverwijder (salbutamol) en controleren we de longfunctie met een blaastest. Het onderzoek duurt maximaal een ½ uur.

'Stikstofmonoxide (NO) meting' is een onderzoek waarbij een bepaald bestanddeel (NO) in uw uitademingslucht wordt gemeten. U wordt gevraagd diep in te ademen en via een mondstuk gedurende 20 seconden langzaam uit te blazen. De uitademingslucht wordt geanalyseerd op de hoeveelheid NO, hetgeen mogelijk een maat is voor ontsteking in de luchtwegen. Het is een weinig belastend onderzoek.

Tenslotte vragen wij u om elke 3 maanden gedurende twee weken een dagboek bij te houden. In dit dagboek noteert u uw luchtwegklachten en de uitslagen van een blaastest.

<u>Belasting en eventuele risico's</u>

Het onderzoek vergt een grote tijdsinvestering. Wij verzoeken u of u elf keer in 2,5 jaar het ziekenhuis wil bezoeken waarvan u drie keer een volledig longonderzoek zal ondergaan. De meeste onderzoeken (bijvoorbeeld de longfunctiemeting) worden normaal ook uitgevoerd wanneer een patiënt begeleid wordt door een longarts. Onderzoeken die voor patiënten met COPD buiten de routine vallen zijn het invullen van vragenlijsten, een 6 minuten looptest, bronchoscopie, sputuminductie en meting van NO in de uitademingslucht.

Op dit moment gebruiken veel patiënten met COPD voor korte of langere tijd inhalatiesteroïden. Tot de dag van vandaag is het nog niet duidelijk of het ziekteproces van COPD beïnvloedt wordt door het gebruik van inhalatiesteroïden. Wij onderzoeken of de medicijnen bij sommige patiënten beter werken dan bij andere en of de medicijnen op de lange termijn minder goed zijn voor patiënten met COPD. Doordat u inhalatiesteroïden inhaleert komt het geneesmiddel direct in uw luchtwegen terecht. Door deze directe manier van toediening veroorzaakt het bijna geen bijwerkingen. In sommige gevallen kan heesheid of een schimmelinfectie in mond- en keelholte optreden. Dit kunt u tegengaan door direct na het inhaleren even uw mond te spoelen met water. Mocht u kort na inhalatie merken dat uw benauwdheid plotseling erger wordt, dan moet u direct de behandeling staken en uw arts raadplegen. U mag (indien van toepassing) geen kinderen krijgen tijdens het onderzoek. Als u dit toch van plan bent of vermoedt dat u zwanger bent, moet u contact opnemen met een van de onderzoekers.

De bronchoscopie is een veilige en routine verrichting die weinig risico met zich meebrengt. Deze wordt door de meest ervaren longartsen van het LUMC verricht. Onmiddellijk na de bronchoscopie kunt u enkele vlekjes bloed ophoesten en een licht zere keel hebben gedurende 24 uur. U kunt kortdurend temperatuurverhoging krijgen. Hiertegen zal paracetamol afdoende zijn. Ook kunt u zich na de bronchoscopie een beetje duizelig voelen. In dit geval dient u wat ventolin te inhaleren. De arts zal ervoor zorgen dat u zich goed herstelt na de bronchoscopie en zal u instrueren wat u moet doen indien een probleem zich voordoet nadat u naar huis bent gegaan. In verband met de verdoving die voor dit onderzoek krijgt adviseren wij u deze dag geen motorvoertuig te besturen.

Andere mogelijke risico's of ongemakken die u tijdens het onderzoek kunt ervaren zijn:

- het maken van een röntgenfoto en hartfilmpjes
- het afnemen van bloedmonsters.
- de procedure van het ophoesten van slijm die eerder is beschreven.

Gedurende het gehele onderzoek wordt u begeleid door de arts-onderzoekers.

Goedkeuring

Het onderzoek is goedgekeurd door de directie en de Commissie Medische Ethiek van het LUMC te Leiden en het AZG van Groningen.

Vertrouwelijkheid

Gedurende het onderzoek worden er medische gegevens verzameld op verschillende formulieren. Op deze formulieren wordt uw naam en adres niet genoemd, maar er wordt een code gebruikt. Alleen uw arts weet welke code bij welke patiënt hoort. Het is van groot belang dat de resultaten van het onderzoek juist worden weergegeven. Daarom kan het voorkomen dat tijdens het onderzoek een bevoegde afgevaardigde van overheidsinstanties de gegevens komt controleren. Eventueel kan ook een bevoegde afgevaardigde van een van de sponsors (Glaxo Wellcome) de gegevens komen controleren. De leider van het onderzoek is er verantwoordelijk voor dat op vertrouwelijke wijze met uw gegevens wordt omgegaan.

Indien u tijdens het onderzoek problemen ondervindt met betrekking tot het onderzoek of de begeleiding, of indien u vragen wilt stellen aan een arts die niet direct bij het onderzoek betrokken is kunt u contact opnemen met:

Dr. E. Bel, Longarts
Afdeling Longziekten, LUMC
071-5262950

Kosten en verzekering

Alle geneesmiddelen, procedures, testen en bezoeken voor deze studie zijn gratis. Uw reiskosten worden in redelijkheid vergoed. De firma die de medicijnen heeft ontwikkeld (Glaxo Wellcome) heeft een speciale verzekering afgesloten voor deze studie (bijlage). Als u vindt dat u schade heeft ondervonden van het onderzoek waaraan u meedoet (of heeft meegedaan) kunt u het beste met uw arts bespreken hoe het met de verzekering is geregeld.

Vrijwilligheid van deelname

Uw medewerking aan dit onderzoek is vrijwillig. Als u toestemming geeft om aan dit onderzoek mee te doen, heeft u te alle tijde de vrijheid om op die beslissing terug te komen. U hoeft hier geen verklaring voor te geven. Het wel of niet meedoen heeft op geen enkele wijze gevolgen voor uw verdere behandeling of de vertrouwenshouding met uw arts.
Ook uw behandelend arts kan uw deelname aan het onderzoek stopzetten als hij of zij vindt dat dit in uw situatie beter is. Hij of zij bespreekt dat dan met u. Neemt u rustig enige bedenktijd voordat u beslist of u meedoet of niet. U kunt deze informatie dan nog eens bespreken met uw partner, familie, huisarts of anderen.

Wie kunt u bellen voor informatie?

Indien u nu of tijdens het onderzoek verdere vragen of zorgen heeft, hoeft u niet te aarzelen om contact op te nemen met de artsen T. Lapperre en J. Stroband.

Naam onderzoeker: T. Lapperre
Functie: arts-onderzoeker
Afdeling: Longziekten
Telefoonnummer: 071-5263261

Naam onderzoeker: J. Stroband
Functie: arts-onderzoeker
Afdeling: Huisartsgeneeskunde
Telefoonnummer: 071-5275350

<u>BIJLAGE bij wetenschappelijk onderzoek:</u>
<u>Verbetering van de behandeling bij patiënten met COPD</u>
protocolnummer (SER9804, GLUCOLD)

Tekst *verplichte schadeverzekering proefpersonen* in het kader van WMO t.b.v. patiënteninformatiebrief.

De *Wet medisch-wetenschappelijk onderzoek met mensen* bepaalt dat voor onderzoek met mensen een schadeverzekering dient te zijn afgesloten. Overeenkomstig deze bepalingen is er ook voor dit onderzoek een verzekering afgesloten die de door het onderzoek veroorzaakte schade door dood of letsel van de proefpersoon dekt.

- Het betreft zowel schade die zich tijdens de deelname van de proefpersoon aan het onderzoek openbaart als schade die zich openbaart en gemeld is binnen 5 jaar na deelname aan het onderzoek.
- Het bedrag waarvoor de verzekering is afgesloten bedraagt fl. 1.000.000,- per proefpersoon en fl. 15.000.000,- voor het gehele onderzoek.

De verzekering biedt geen dekking voor schade

- die zich bij nakomelingen openbaart als gevolg van een nadelige inwerking van het onderzoek op het genetisch materiaal van de proefpersoon;
- door aantasting van de gezondheid van de proefpersoon die zich ook zou hebben geopenbaard wanneer de proefpersoon niet aan het onderzoek had deelgenomen;
- waarvan op grond van de aard van het onderzoek zeker of nagenoeg zeker was dat deze zich zou voordoen;
- die het gevolg is van het niet of niet volledig opvolgen van aanwijzingen en instructies door de proefpersoon, indien de proefpersoon daar althans toe in staat is.

Om, in geval van vermeende schade als gevolg van het onderzoek, aanspraak te kunnen maken op schadevergoeding, dient deze rechtstreeks gemeld te worden bij de verzekeraar.

Naam verzekeraar:	Royal & SunAlliance Global & Large Risks
Adres verzekeraar:	Postbus 413<br>3000 AK Rotterdam
Contactpersoon:	J.L. Schürmann
Telefoonnummer:	010-400 7916
Faxnummer:	010-413 7622

U dient bij het melden van schade het volgende te vermelden:

- de titel van het onderzoek: ………………
- het protocolnummer: ……………………
- het referentienummer: 011 0007571/HOL.
- uw persoonsgegevens (naam, adres, telefoonnummer) opdat de verzekeraar u kan bereiken voor nadere informatie.
- de naam van de behandelend arts tijdens het onderzoek en indien van toepassing de naam van het ziekenhuis waar u behandeld bent.

TOESTEMMINGSVERKLARING
VOOR DEELNAME
AAN WETENSCHAPPELIJK ONDERZOEK

naar effecten van medicijngebruik bij mensen met chronisch obstructieve longziekten (COPD).

Groningen
Leiden
Universities
Corticosteroids in
Obstructive
Lung
Disease

Protocolnummer van het onderzoek:

Ondergetekende verklaart hierbij het volgende:

- Ik heb de bijbehorende informatiebrief gelezen en begrepen;
- Ik ben voldoende geïnformeerd over het doel en de procedures van het onderzoek;
- Ik heb voldoende tijd gehad om over deelname aan het onderzoek te beslissen;
- Ik heb de gelegenheid gehad om mijn vragen te bespreken met de arts;
- Ik heb begrepen dat extra bloed wordt afgenomen ten behoeve van eventueel toekomstig wetenschappelijk onderzoek, zoals bijvoorbeeld erfelijkheidsonderzoek, zonder dat ik daarover word geïnformeerd;
- Ik geef toestemming aan bevoegde afgevaardigden van overheidsinstanties en eventueel een van de sponsors om mijn medische gegevens die betrekking hebben op het onderzoek te controleren;
- Ik heb begrepen dat ik mij te allen tijde en dus ook voor of na de bronchoscopie kan terugtrekken uit het onderzoek zonder opgaaf van redenen en zonder dat dit gevolgen heeft voor mijn verdere behandeling;
- Zoals afgesproken vul ik na de eerste bronchoscopie een vragenlijst in over deze ingreep en beslis nogmaals over verdere deelname aan dit project. Op dit formulier zal ik aangeven of ik verder wil deelnemen aan de studie.
- Ik heb begrepen dat in dit onderzoek drie bronchoscopieën worden verricht, dat de artsen mij voor elke bronchoscopie nadrukkelijk vragen of ik deze ingreep wil ondergaan en een apart toestemmingsformulier wil tekenen.
- (Indien van toepassing) ik heb begrepen dat ik tijdens het onderzoek niet zwanger mag zijn of worden.

Ik geef op vrijwillige basis toestemming tot deelname aan dit onderzoek

Achternaam en voorletters van de patiënt..
(in blokletters a.u.b.)
Geboortedatum..

Handtekening patiënt... .Datum ....

Ondergetekende verklaart dat de hierboven genoemde persoon zowel mondeling als schriftelijk over bovenvernoemd onderzoek is geïnformeerd Hij/zij verklaart tevens dat een voortijdige beëindiging van de deelname door de bovengenoemde persoon, van geen enkele invloed al zijn op de zorg die hem of haar toekomt.

Naam en voorletters van de arts ....
(in blokletters a.u.b.)
functie:..
Handtekening arts.. Datum ....

TOESTEMMINGSVERKLARING

VOOR BRONCHOSCOPIE

in het onderzoek naar effecten van medicijngebruik bij mensen met chronisch obstructieve longziekten (COPD).

Groningen
Leiden
Universities
Corticosteroids in
Obstructive
Lung
Disease

Protocolnummer van het onderzoek:

Ondergetekende verklaart hierbij het volgende:

- Ik ben voldoende geïnformeerd over het doel en de procedure van een bronchoscopie, en ben op de hoogte van de mogelijke risico's en ongemakken die de bronchoscopie mogelijk met zich mee kunnen brengen;
- Ik heb voldoende tijd gehad om over deelname aan deze bronchoscopie na te denken;
- Ik heb de gelegenheid gehad om mijn vragen omtrent de bronchoscopie te bespreken met de arts of mijn huisarts;
- Ik heb begrepen dat is afgesproken dat ik na de eerste bronchoscopie een vragenlijst over deze ingreep zal invullen en nogmaals moet beslissen over verdere deelname aan dit project. Op dit formulier zal ik aangeven of ik verder wil deelnemen aan de studie.
- Ik heb begrepen dat in dit onderzoek drie bronchoscopieën worden verricht, dat de artsen mij voor elke bronchoscopie nadrukkelijk vragen of ik deze ingreep wil ondergaan, of ik een toestemmingsformulier wil tekenen en een vragenlijst wil invullen.
- Ik heb begrepen dat ik mij te allen tijde en dus ook voor of na de bronchoscopie kan terugtrekken uit het onderzoek zonder opgaaf van redenen en zonder dat dit gevolgen heeft voor mijn verdere behandeling;

Ik geef op vrijwillige basis toestemming tot deelname aan de bronchoscopie

Achternaam en voorletters van de patiënt..
(in blokletters a.u.b.)

Geboortedatum..

Handtekening patiënt... .Datum ....

Ondergetekende verklaart dat de hierboven genoemde persoon zowel mondeling als schriftelijk over de bronchoscopie is geïnformeerd Hij/zij verklaart tevens dat een voortijdige beëindiging van de deelname door de bovengenoemde persoon, van geen enkele invloed al zijn op de zorg die hem of haar toekomt.

Naam en voorletters van de arts ....
(in blokletters a.u.b.)

Functie:...

Handtekening arts
Datum

VRAGENLIJST

BRONCHOSCOPISCH ONDERZOEK

Groningen
Leiden
Universities
Corticosteroids in
Obstructive
Lung
Disease

Zoals afgesproken is hier de vragenlijst over de bronchoscopie. Deze vragenlijst vormt voor u de basis om verder mee te doen aan het wetenschappelijk onderzoek naar de behandeling van patiënten met COPD. Na het invullen kunt u rustig nadenken over verdere deelname aan de studie. Wij zullen contact met u opnemen en u kunt dan met ons bespreken welke beslissing u heeft genomen. Indien u deel wilt nemen aan het vervolg van het onderzoek zullen wij u vragen nogmaals een algemeen toestemmingsformulier te ondertekenen.

Wilt u de onderstaande vragen beantwoorden door een kruisje te zetten in één van de vakjes, waarmee u aangeeft of u het al dan niet met deze stelling eens bent. Na het invullen kunt u de vragenlijst in de bijgevoegde retourenvelop voegen en op de bus doen.

Voorbeeld:

0. Het was vandaag mooi weer.

helemaal niet mee eens -3 -2 -1 0 1 2 3 helemaal mee eens
|----|----|----|----|----|----|--**X**-|

Hopelijk was dit een juist antwoord!

Vragenlijst:

1 Ik vind een bronchoscopie een belastend onderzoek.

helemaal niet mee eens -3 -2 -1 0 1 2 3 helemaal mee eens
|----|----|----|----|----|----|----|

2 Een bronchoscopie vind ik meer belastend dan de inhalatie-provokatietest.

helemaal niet mee eens -3 -2 -1 0 1 2 3 helemaal mee eens
|----|----|----|----|----|----|----|

3 Ieder ander zou ik een bronchoscopie afraden.

helemaal niet mee eens -3 -2 -1 0 1 2 3 helemaal mee eens
|----|----|----|----|----|----|----|

4 Ik heb veel moeten hoesten tijdens de bronchoscopie.

helemaal niet mee eens -3 -2 -1 0 1 2 3 helemaal mee eens
|----|----|----|----|----|----|----|

5 Ik heb erg tegen de bronchoscopie opgezien.

helemaal niet mee eens -3 -2 -1 0 1 2 3 helemaal mee eens
|----|----|----|----|----|----|----|

6. Ik was erg kortademig tijdens de bronchoscopie.

helemaal niet mee eens -3 -2 -1 0 1 2 3 helemaal mee eens
|----|----|----|----|----|----|----|

7. Ik wist van tevoren precies hoe de bronchoscopie zou verlopen.

helemaal niet mee eens -3 -2 -1 0 1 2 3 helemaal mee eens
|----|----|----|----|----|----|----|

8. Dit was een vervelend onderzoek. helemaal niet mee eens -3 -2 -1 0 1 2 3 |----|----|----|----|----|----|----| helemaal mee eens

9. Een volgende keer zou ik deze bronchoscopie weer doen. helemaal niet mee eens -3 -2 -1 0 1 2 3 |----|----|----|----|----|----|----| helemaal mee eens

10. De rest van de dag heb ik weinig hinder van de bronchoscopie ondervonden. helemaal niet mee eens -3 -2 -1 0 1 2 3 |----|----|----|----|----|----|----| helemaal mee eens

Tenslotte willen we u nog een paar vragen stellen over het beloop van de bronchoscopie, om zonodig dingen te verbeteren:

11. Ik vond de informatie vooraf duidelijk. helemaal niet mee eens -3 -2 -1 0 1 2 3 |----|----|----|----|----|----|----| helemaal mee eens

12. Het bekijken van de video vooraf was nuttig. helemaal niet mee eens -3 -2 -1 0 1 2 3 |----|----|----|----|----|----|----| helemaal mee eens

13. Met de verkregen informatie was ik goed op de bronchoscopie voorbereid. helemaal niet mee eens -3 -2 -1 0 1 2 3 |----|----|----|----|----|----|----| helemaal mee eens

14. Ik vond de begeleiding tijdens de bronchoscopie goed. helemaal niet mee eens -3 -2 -1 0 1 2 3 |----|----|----|----|----|----|----| helemaal mee eens

15 De nazorg na het onderzoek was goed. helemaal -3 -2 -1 0 1 2 3 helemaal

niet mee eens |----|----|----|----|----|----|----| mee eens

17 Wat vond u het vervelendste onderdeel van de bronchoscopie?

18. Heeft u nog opmerkingen of suggesties betreffende de bronchoscopie?

Ik geef op vrijwillige basis toestemming tot verdere deelname aan dit onderzoek: ja / nee*

Achternaam en voorletters van de patiënt..

Handtekening patiënt.. .Datum ....

* doorhalen wat niet van toepassing is
