# Supplementary material for: Prediction of Long-Term Benefits of Inhaled Steroids by Phenotypic Markers in Moderate-to-Severe COPD: A Randomized Controlled Trial
Source: PLoS One. 2015 Dec 10;10(12):e0143793. doi: 10.1371/journal.pone.0143793 (PMC4699453; doi:10.1371/journal.pone.0143793)
Supplement: S1 Protocol Amendment — (PDF) [file pone.0143793.s003.pdf]

LUMC

LEIDS UNIVERSITAIR MEDISCH CENTRUM

Afdeling Commissie Medische Ethiek/ H1-Q

Webpagina http://cme.clinicalresearch.nl
Telefoon 071-526 3241 fax 071-526 69 63
e-mail, secr. C.E.L.de_Win@lumc.nl
e-mailsecretaris W.Kool @lumc.nl

Prof. dr. P.J. Sterk

Afdeling Longziekten

Adres **C2-P (alhier)**

onze referentie **Protocolnummer P211/98/**YR/ib

Datum 24 maart 2003
Onderwerp **Goedkeuring amendement nr. 2**

Bij alle correspondentie en informatie Protocolnummer vermelden!!

Zeer geachte heer/mevrouw,

In antwoord op uw brief van 7 maart 2003 met daarbij gevoegd amendement nr. 2 d.d. 5 maart 2003 inzake onderzoeksprotocol P211/98 "GLUCOLD-STUDIE - Modification of disease outcome in COPD: prediction of long-term clinical course", bericht de Commissie Medische Ethiek u dat zij geen bezwaren heeft tegen de beschreven wijzigingen.

In het vertrouwen u hiermee voldoende te hebben geïnformeerd,

Met vriendelijke groet,

Mw. Mr. Y.M. Reidsma
Secretaris

LUMC

LEIDS UNIVERSITAIR MEDISCH CENTRUM

Afdeling Commissie Medische Ethiek/ H1-Q

Webpagina http://cme.clinicalresearch.nl
Telefoon 071-526 3241 fax 071-526 69 63
e-mail, secr. C.E.L.de_Win@lumc.nl
e-mailsecretaris W.Kool @lumc.nl

De hooggeleerde heer
Prof. dr. P.J. Sterk

Afdeling Longziekten

Adres **C2-P, alhier**

onze referentie **Protocolnummer P211/98**/WK/wk

Datum 16 juli 2003
Onderwerp **Goedkeuring amendement**

Bij alle correspondentie en informatie Protocolnummer vermelden!!

Zeer geachte heer,

Hierbij bericht de Commissie Medische Ethiek u dat zij uw brief van 24 juni 2003 met daarbij gevoegd een amendement d.d. 24-06-2003 en bijbehorende proefpersoneninformatie d.d. 13-6-2003 inzake onderzoeksprotocol P211/98 "GLUCOLD-STUDIE - Modification of disease outcome in COPD: prediction of long-term clinical course", in goede orde heeft ontvangen en dat zij geen bezwaren heeft tegen de beschreven uitbreiding van het onderzoek met een CT-scan.

Vertrouwend u hiermee voldoende te hebben geïnformeerd.

Met vriendelijke groet,
namens de Commissie Medische Ethiek,

W Kool.

Mw. mr. W. Kool
Secretaris
